# Early deficits in an in vitro striatal microcircuit model carrying the Parkinson’s GBA-N370S mutation

**DOI:** 10.1038/s41531-024-00694-2

**Published:** 2024-04-12

**Authors:** Quyen B. Do, Humaira Noor, Ricardo Marquez-Gomez, Kaitlyn M. L. Cramb, Bryan Ng, Ajantha Abbey, Naroa Ibarra-Aizpurua, Maria Claudia Caiazza, Parnaz Sharifi, Charmaine Lang, Dayne Beccano-Kelly, Jimena Baleriola, Nora Bengoa-Vergniory, Richard Wade-Martins

**Affiliations:** 1 Oxford Parkinson’s Disease Centre and Department of Physiology, Anatomy and Genetics, University of Oxford, South Park Road, Oxford, OX1 3QU UK; 2 Kavli Institute for Neuroscience Discovery, University of Oxford, Dorothy Crowfoot Hodgkin Building, South Park Road, Oxford, OX1 3QU UK; 3 Aligning Science Across Parkinson’s (ASAP) Collaborative Research Network, Chevy Chase, MD 20815 USA; 4 Nuffield Department of Medicine (NDM), University of Oxford, Henry Wellcome Building for Molecular Physiology, Old Road, Oxford, OX3 7BN UK; 5 Achucarro Basque Center for Neuroscience, Leioa, Spain; 6 Ikerbasque—Basque Foundation for Science, Bilbao, Spain; 7 University of the Basque Country (UPV/EHU), Department of Neuroscience, Leioa, Spain

**Keywords:** Synaptic transmission, Cellular neuroscience

## Abstract

Understanding medium spiny neuron (MSN) physiology is essential to understand motor impairments in Parkinson’s disease (PD) given the architecture of the basal ganglia. Here, we developed a custom three-chambered microfluidic platform and established a cortico-striato-nigral microcircuit partially recapitulating the striatal presynaptic landscape in vitro using induced pluripotent stem cell (iPSC)-derived neurons. We found that, cortical glutamatergic projections facilitated MSN synaptic activity, and dopaminergic transmission enhanced maturation of MSNs in vitro. Replacement of wild-type iPSC-derived dopamine neurons (iPSC-DaNs) in the striatal microcircuit with those carrying the PD-related *GBA-N370S* mutation led to a depolarisation of resting membrane potential and an increase in rheobase in iPSC-MSNs, as well as a reduction in both voltage-gated sodium and potassium currents. Such deficits were resolved in late microcircuit cultures, and could be reversed in younger cultures with antagonism of protein kinase A activity in iPSC-MSNs. Taken together, our results highlight the unique utility of modelling striatal neurons in a modular physiological circuit to reveal mechanistic insights into *GBA1* mutations in PD.

## Introduction

The striatum is comprised of 90% medium-sized spiny neurons (MSNs) and represents the primary input nucleus of the basal ganglia^1^. MSNs receive converging glutamatergic and dopaminergic input from the cortex/thalamus and midbrain, respectively, forming a presynaptic dyad intrinsically regulating MSN functions. Striatal dysfunction plays a key role in the motor features of Parkinson’s disease (PD), although the exact underlying mechanisms remain under intense study with divergent findings^2–4^. The consensus from animal models of PD suggests that the characteristic preferential degeneration of substantia nigra *pars compacta* (SNc) dopaminergic neurons (DaNs) results in aberrant striatal activity patterns which subsequently alter the balanced output of the basal ganglia governing motor behaviour^5^. However, much less is known about how pathological DaNs carrying PD-related mutations impact function of the downstream MSNs. Currently available two-dimensional (2D) models of iPSC-derived MSN monoculture^6–8^, and 3D organoids^9^ have not included DaNs while the very recent three-dimensional midbrain-striatum-cortex assembloid^10^ did not investigate the role of PD mutations in its circuit.

*GBA1* mutations are major genetic risk factors for PD with the N370S (the c.1226 A > G) variant being one of the most common worldwide^11^. Multiple previous studies have proposed autonomous cellular dysfunction of DaNs harbouring the *GBA-N370S* mutation and a growing body of literature suggests that this mutation leads to failure of the mitochondria and the endolysosomal pathway^12–14^. However, how the mutant DaNs contribute towards physiological perturbation of the striatal output and the eventual motor impairment in PD is still unknown.

Microfluidic neuronal culture technology has tremendously assisted efforts in modelling and understanding biological systems, primarily enabling custom and precise control of neurite isolation^15,16^ as well as orchestrated neuronal circuitry^17,18^. In this work we have established a microfluidic platform comprising an all iPSC-derived striatal presynaptic dyad to recapitulate the endogenous connectivity of cortical and dopamine neurons onto MSNs. This setup is permissive to long-term cultures of up to 100 days and allows for the investigation of temporal changes in neuronal physiology. With this system we have dissected the importance of dual presynaptic glutamatergic and dopaminergic inputs in facilitating electrophysiological maturation of in vitro MSNs and in doing so have found a potential effect of the *GBA-N370S* genetic mutation in DaNs on the electrophysiological properties of MSNs in vitro.

## Results

### Generation of functional iPSC-derived medium spiny neurons (iPSC-MSNs)

**Fig. 1:**
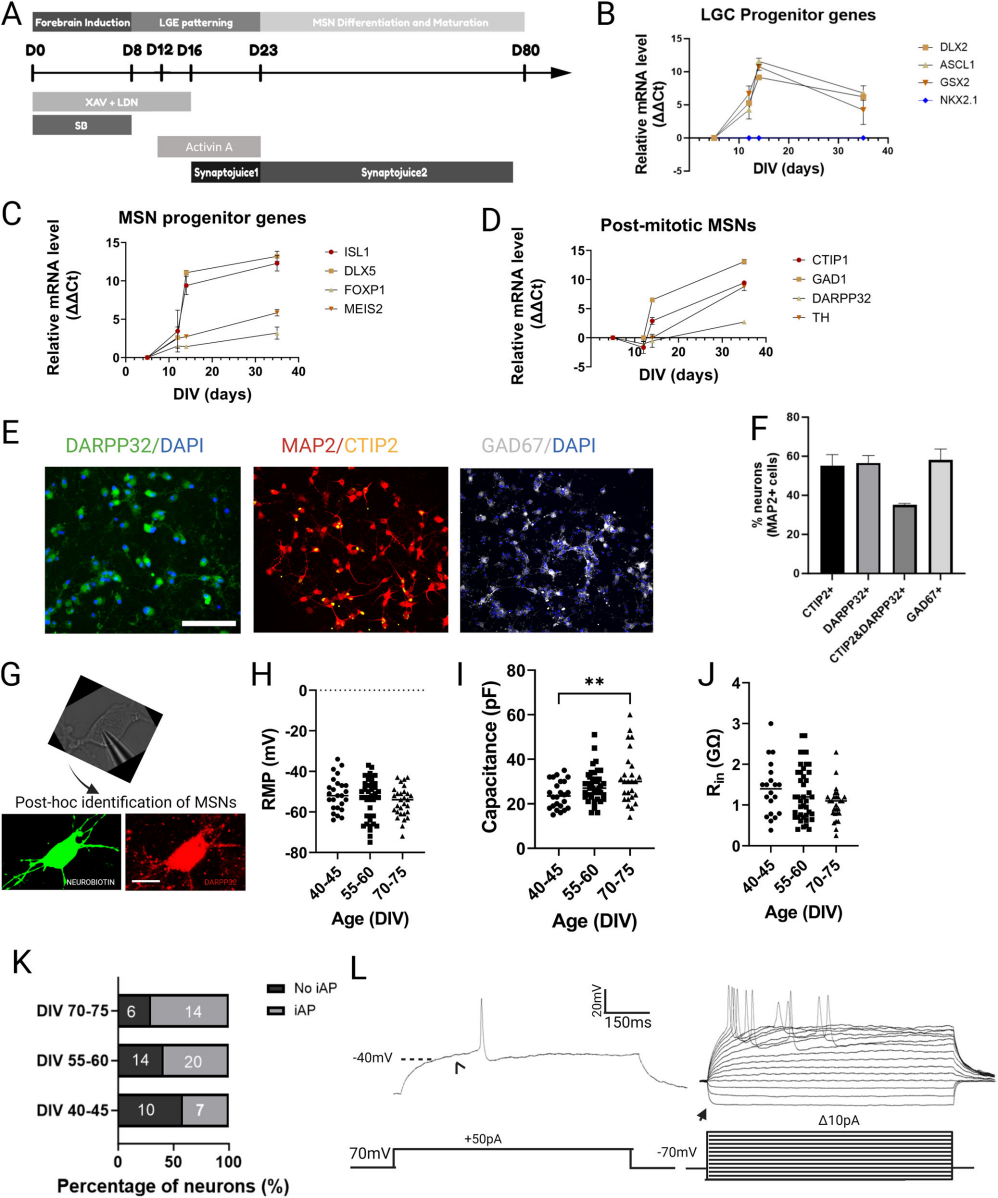
Generation and characterisation of iPSC-MSNs. **A** Differentiation schematic of medium spiny neurons (MSNs) from iPSCs. Temporal expression of selected markers of LGE progenitors (**B**), MSN progenitors (**C**) and post-mitotic MSNs (**D**) from DIV 0 till 35. Data are presented as mean ± sem. **E** Representative fluorescent images of DIV 40 MSN cultures expressing MAP2 (red), CTIP2 (yellow), DARPP32 (green), GAD67 (grey) and DAPI (blue), scale bar = 100 µm, and (**F**) quantification of their expression in percentage term at DIV 40–45. Data are presented as mean ± sem. **G** Whole-cell patch-clamp recording and subsequent post-hoc identification of neurobiotin-filled DARPP32+ neurons for electrophysiological analysis. Scale bar = 10 µm. Passive electrophysiological properties including (**H**) Resting membrane potential (RMP), (**I**) cell capacitance, and (**J**) input resistance (R_in_) of iPSC-MSNs over time from DIV 40–75, *n* = 20–43 recording MSNs per condition, each dot represents a single recording neuron, data are presented as mean ± SD, One-way ANOVA corrected with Bonferroni post-hoc test, **p < 0.01, ns *p* > 0.05. **K** Number of MSNs that displayed evoked action potential upon current injection (iAP) and MSNs that did not (no iAP) over time. **L** Representative traces of evoked APs (iAP) upon injection of depolarising currents. *N* = 3–4 differentiation experiments of 3 iPSC lines.

We first applied a defined cocktail of small molecules to recreate the temporal in vivo development of MSNs to reflect their telencephalic and lateral ganglionic eminence (LGE) lineage (Fig. 1A). To induce neuroectodermal development at the start of the differentiation, we used modulators of SMAD, namely LDN and SB43154^19^, and an inhibitor of the WNT signalling pathway (XAV)^20^. Activin A, which was independently reported to enhance dorsal striatal patterning^7,9^, was introduced from DIV 12-23 to promote LGE identity. Activin A increased the expression of selected transcripts specific for LGE and MSN progenitors two days post treatment (Supplementary Fig. 1A) as well as the proportion of post-mitotic neurons co-expressing DARRP32 and CTIP2 on DIV 40 (Supplementary Fig. 1B). Temporal examination of transcript abundance of LGE and MSN progenitor markers using RT-qPCR suggested gradual patterning of iPSCs towards MSNs reminiscent of their in vivo developmental trajectory (Fig. 1B–D). LGE-specific genes such as *DLX2, ASCL1*, and *GSX2* were enriched at early timepoints and this enrichment was subsequently reduced in post-mitotic MSNs (Fig. 1B). Meanwhile, expression of selected markers of MSN progenitors including *ISL1, DLX5, FOXP2*, and *MEIS2* increased over time and remained abundant in post-mitotic MSN cultures (Fig. 1C). *CTIP2, GAD1*, and *DARPP32*, which are indicative of post-mitotic MSNs, were virtually absent in early cultures and only subsequently expressed in great abundance from DIV 14 (Fig. 1D). Notably, transcript expression of *NKX2*.1, the medial ganglionic eminence marker, was barely detectable throughout the culture development (Fig. 1B), indicating a lack of MGE identity and strong LGE enrichment in the patterning. Expression of specific markers for cortical neurons such as TBR1 and SATB2 was absent in the resulting MSN cultures (Supplementary Fig. 2A). Tyrosine hydroxylase (*TH*) transcript (Fig. 1D) and TH protein (Supplementary Fig. 2B, C) were detected in a small proportion of the post-mitotic cultures (7.05% ± 1.73). Interestingly, the detected TH-positive neurons were FOXA2-negative, implying the presence of potentially non-MSNs, TH-expressing interneurons of striatal identity which are not of floor-plate origin.

More than 50% of MAP2+ iPSC-MSNs at DIV 40 expressed CTIP2, DARPP32, or GAD67 and up to 35% co-expressed both canonical markers CTIP2 and DARPP32 (Fig. 1E, F). We also observed expression of selected markers specific to either the direct- or indirect-pathway MSNs (Supplementary Fig. 3). Immunostaining confirmed expression of MSN subtype-specific neurotransmitter and neurotransmitter precursors including PENK, PDYN and Substance P at DIV 50 (Supplementary Fig. 3A–C). Quantification of GAD67-positive GABAergic neurons co-expressing either PENK or PDYN or Substance P suggested the more dominant presence of direct pathway MSNs, which are mostly PDYN-positive (22.21% ± 8.55) intermingled with a modest population of Substance P-positive neurons (1.16 ± 0.85), as well as a more minor presence of indirect pathway MSNs expressing PENK (0.72% ± 0.84) in our iPSC-MSN cultures (Supplementary Fig. 3D).

We next performed whole cell patch-clamping to examine functional properties of DARPP32-positive iPSC-MSNs (Fig. 1G). Our iPSC-MSNs displayed hyperpolarised resting membrane potential (DIV 40–45: −51.2 ± 8.2 mV, *n* = 25 neurons; DIV 55–60: −53.3 ± 9.7, *n* = 43 neurons; DIV 70–75: −55.1 ± 7.3 mV, *n* = 29 neurons), large cell capacitance (DIV 40–45: 24.0 ± 6.2 pA, *n* = 24 neurons; DIV 55–60: 28 ± 7.2pA, *n* = 42 neurons; DIV 70–75: 31.6 ± 11.8 pA, *n* = 26 neurons), and large input resistance (R_in_) (DIV 40–45: 1.4 ± 0.7GΩ, *n* = 20 neurons; DIV 55–60: 1.3 ± 0.7 GΩ, *n* = 41 neurons; DIV 70–75: 1.0 ± 0.4 GΩ, *n* = 27 neurons) (Fig. 1H–J). There was no significant change of these intrinsic properties of MSNs from DIV 40–75 (RMP: *p* = 0.265; R_in_: *p* = 0.09; all were analysed with one-way ANOVA with Bonferroni multiple comparison correction test) with the exception of cell capacitance between DIV 40–45 and DIV 70–75 (*p* = 0.006). iPSC-MSNs did not fire spontaneously but action potentials (APs) were evoked with depolarising current injections, albeit with immature characteristics such as the lack of delayed first spike (arrowhead) (Fig. 1L), low rheobase, small evoked AP amplitude, and low frequency (Supplementary Table 1 and Supplementary Fig. 4). The proportion of functionally active neurons that displayed evoked APs compared to those that were unresponsive increased in late cultures (DIV 40–45: 7/10 neurons; DIV 55–60: 20/14; DIV 70–75: 14/6) (Fig. 1K) without significant improvement in AP specific properties (Supplementary Fig. 4). Together, these data indicated robust generation of molecularly defined and functionally active, albeit immature, iPSC-MSN monocultures that are electrophysiologically stable from DIV 40 to 75.

### In vitro recapitulation of the striatal microcircuit on custom microfluidic devices

Despite the functionally active status of iPSC-MSNs in monocultures, we noted a more depolarised RMP and larger R_in_ in in vitro MSNs relative to the in vivo^21^. We hypothesised that such discrepancy was principally due to the lack of non-autologous synapses that endogenously provide valuable developmental cues to modulate MSN functional maturation.

**Fig. 2:**
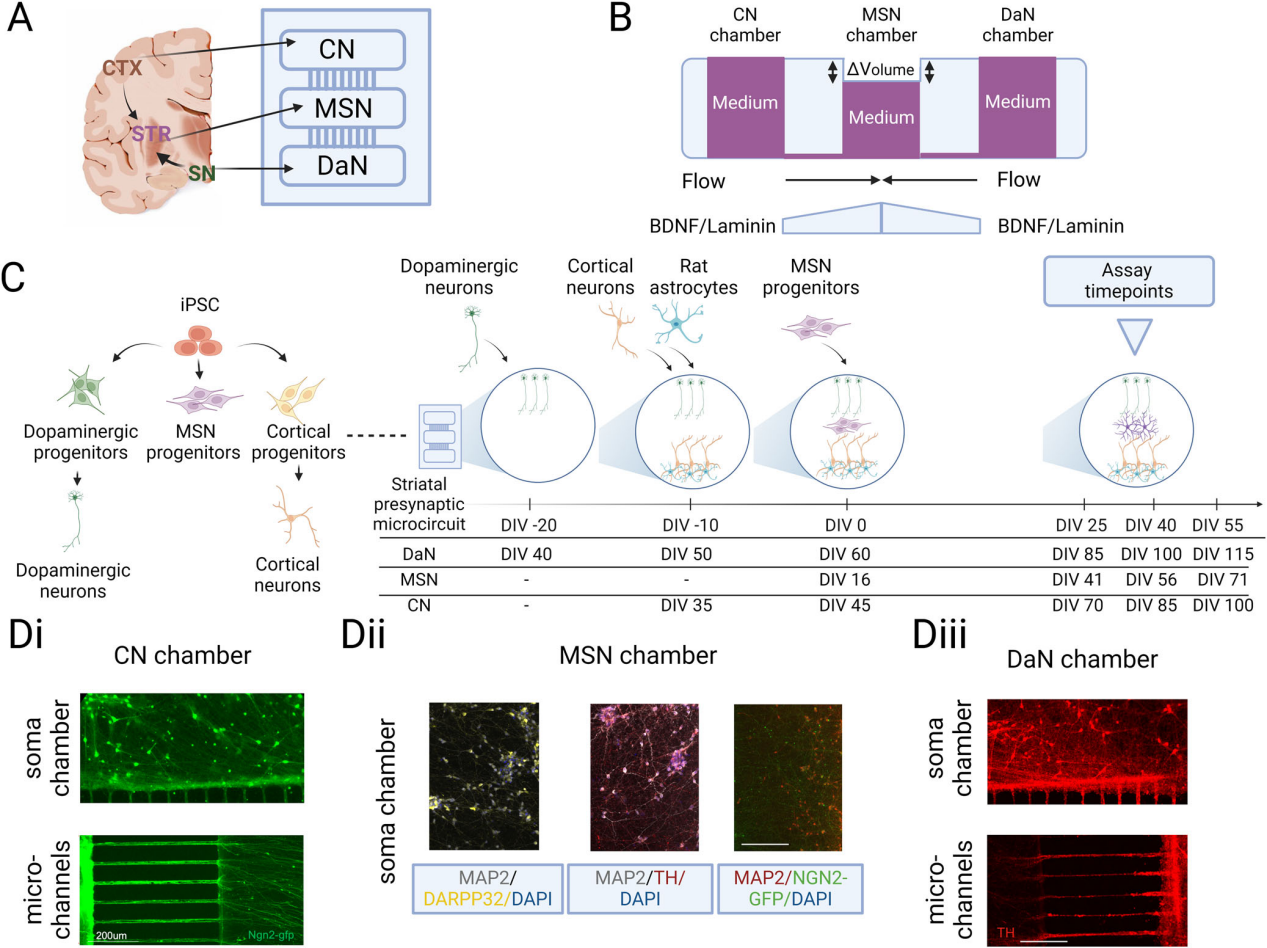
In vitro modelling of the directed striatal presynaptic dyad using microfluidic-based devices. **A** Schematic of custom microfluidic device which mimics orientation of the cortico-striato-nigral circuit. **B** Side view schematics of fluid and growth factor-driven culturing paradigm of the cortico-striato-nigral microcircuit. **C** Schematic of the experimental design concept showing the culturing workflow to reconstruct the directional cortico-striato-nigral microcircuit in vitro. Neuronal subtypes were independently differentiated from iPSC stage to committed progenitors on standard well-plates prior to be sequentially seeded onto the microfluidic devices. Corresponding days in vitro (DIV) of each neuronal subtypes at different plating events and experimental timepoints were included in the timeline. **D** Representative images showing compartmentalisation of (Di) cortical neurons (CNs), (Dii) MSNs and (Diii) dopaminergic neurons (DaNs) in their respective chamber and uni-directional traversing of axons through microchannels.

To address the lack of maturational signalling within MSN monocultures, we reconstructed the striatal microcircuit in vitro mimicking part the complex presynaptic input on MSNs using polydimethylsiloxane-based open three-chambered microfluidic chips. The device consisted of three rectangle chambers (4*10 mm) connected via an array of approximately 120 microgrooves of 5 µm wide, 450 µm long and 10 µm high (Fig. 2A, B). The narrow width of the micro-channels compartmentalised the neuronal soma in its respective chamber while only axons, but not dendrites, can traverse the full 450 µm length of the microchannels to make connections with neurons in the middle chamber (Fig. 2C, D).

DaNs, CNs and MSNs were independently differentiated to committed progenitor state from iPSCs on standard multi-well plates as per our previously reported protocols^22,23^ with slight modifications (see Methods) so as to generate neurons expressing their specific markers and capable of releasing their specific neurotransmitters (Fig. 1 and Supplementary Figs. 5 and 6). Progenitor neurons i.e., DIV 16 iPSC-MSNs, DIV 40 iPSC-DaNs and DIV 35 CNs were sequentially and finally plated into their respective chambers so as to recapitulate the cortico-striato-nigral microcircuit (Fig. 2C, D). Rat astrocytes were co-cultured with CNs in the cortical chamber to promote the neuronal and network maturation of the cortical population^22^. Maintenance of individual neuronal populations in their chambers followed their respective differentiation paradigms for monoculture. DaN and CN axons were physically guided towards the middle MSN chamber via flow driven by differential pressure generated by a volume difference between chambers (Fig. 2A, B). Additionally, we exploited the previous observation that brain-derived growth factor (BDNF) and laminin are selectively critical for axon polarisation and outgrowth^24,25^ by setting up artificial BDNF and laminin gradients across the channels to chemically attract axonal projections from CN and DaN chambers into the middle MSN chambers (Fig. 2B) prior to MSN deposition. Growth of MSN axons travelling out to the DaN and CN chambers was restricted by delayed seeding of iPSC-MSNs until after all microgrooves had been populated with DaN or CN axons (Fig. 2Di, iii). By DIV 0 of the microcircuit culture, iPSC-DaNs (i.e., DIV 60) and iPSC-CNs (DIV 45) had developed into functionally active neurons^22,26^ while their axons had also densely projected into the MSN chamber. In addition to attaining directed connectivity, the culturing schedule in Fig. 2C was also designed to incorporate developmental and technical milestones. The culture was maintained until the experimental timepoints (i.e., DIV 40–70 MSNs) at which three neuronal populations were individually electrically active and stable in their respective monocultures, as reported in our previous work^22,26^. Long-term viability of all three neuronal subtypes within microfluidic devices was confirmed by the relatively stable expression of postmitotic (i.e., MAP2+) neurons within each chamber as well as the constant abundance of neurons expressing respective subtype-specific markers over time (Supplementary Fig. 7). Formation of synapses and pre- and post-synaptic apposition was already observed at our first experimental timepoint, 25 days after MSN replating i.e., DIV 40 iPSC-MSNs (Supplementary Fig. 8).

### Glutamatergic input facilitates synaptic maturation of in vitro MSNs

Cortical and dopaminergic afferents form characteristic presynaptic dyads with MSN dendrites, specifically on MSN dendritic spine heads and necks, respectively^27,28^. Corticostriatal terminals represent the major source of glutamatergic input to regulate striatal GABAergic output while dopamine is thought to exert a modulatory influence on the responsiveness of MSNs to excitatory synaptic signals via the regulation of the cell excitability state^21,29,30^.

**Fig. 3:**
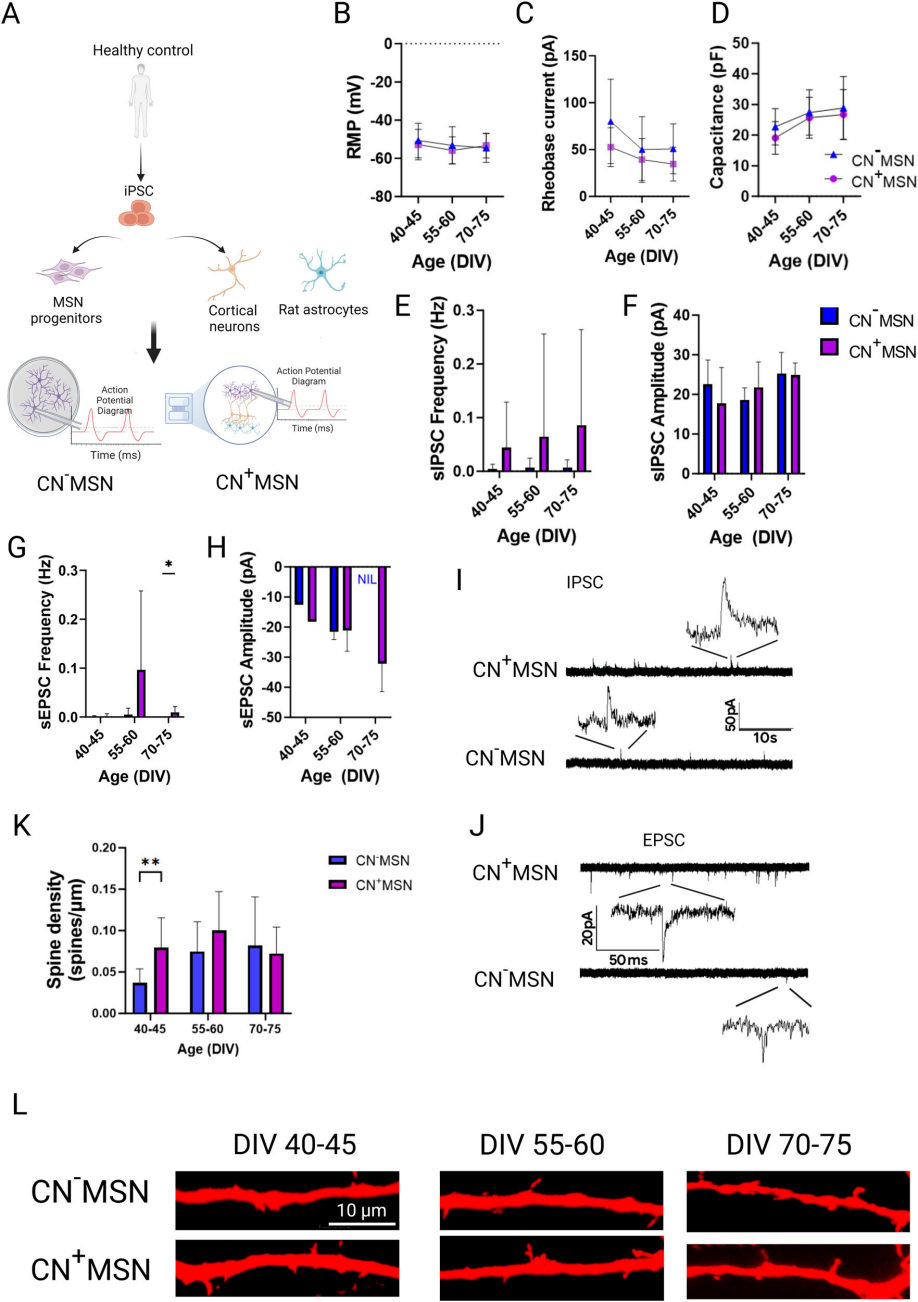
Coculturing iPSC-MSNs with iPSC-CNs improved synaptic activity and dendritic spine morphology of iPSC-MSNs. **A** Schematics illustrating the culturing setup and whole cell patch-clamp recording of iPSC-MSNs in monoculture (CN^−^MSN) or in co-culture with cortical neurons in microfluidic devices (CN^+^MSN). **B** Resting membrane potential (RMP), (**C**) rheobase current, and (**D**) input resistance of CN^-^MSN and CN^+^MSN, *n* = 12–42, 7–27, and 12–42 recording MSNs per condition, respectively. Quantification of spontaneous inhibitory postsynaptic current (sIPSC) (**E**) frequency (**F**) and amplitude over time from DIV 40–75, *n* = 10–37 recording MSNs per condition. Quantification of spontaneous excitatory postsynaptic current (sEPSC) (**G**) frequency and (**H**) magnitude of CN^-^MSN and CN^+^MSN over time from DIV 40–75. *N* = 9-21 recording MSNs per condition. Representative trace of (**I**) IPSC and (**J**) EPSC of CN^-^MSN and CN^+^MSN. **K** Quantification of spine density in CN^−^MSN and CN^+^MSN between DIV 40–75, *n* = 11–29 recording MSNs per condition (**L**) Representative fluorescent images of dendritic spines in neurobiotin-filled DARPP32+ MSNs across three timepoints. All data are presented as mean ± SD, *N* = 3 differentiation experiments of 3 iPSC lines, 2-way ANOVA with Bonferroni post-hoc correction test. *p < 0.05, **p < 0.01.

To examine the physiological role cortical collaterals exerted onto iPSC-MSNs in our in vitro microcircuit, we compared various electrophysiological parameters between iPSC-MSNs grown on coverslips in monoculture (i.e., CN^−^MSN) versus those co-cultured with iPSC-CNs (i.e., CN^+^MSN) on microfluidic devices (Fig. 3A). The modular nature of our culturing platform allows oriented coupling of only CNs and MSNs without DaNs (i.e., CN^+^MSN) following the same culturing timeline (Fig. 2C) so as to dissect the CN-effect on in vitro MSN electrophysiology. We found no differences in RMP across time or between culture conditions over DIV 40–70 (RMP: culturing condition *p* > 0.05; DIV *p* > 0.05). No significant effect of culturing conditions was observed for both cell capacitance and rheobase current at all three timepoints (CN^−^MSN versus CN^+^MSN: all *p* > 0.05) (Fig. 3B–D). CN^+^MSN also displayed no differences in spontaneous inhibitory postsynaptic current (sIPSCs), both in terms of frequency and amplitude of detected sIPSC events (all *p* > 0.05) (Fig. 3E, F, I). However, coculture with CNs exerted significant effect on frequency of spontaneous excitatory postsynaptic currents (sEPSCs) in MSNs. Only 4 out of 34 recording neurons in CN^-^MSN cultures across all three timepoints showed sEPSC activity (DIV 40–45: *n* = 1, DIV 55–60: *n* = 3, and DIV 70–75: *n* = 0). Meanwhile, sEPSCs were much more likely to occur in CN^+^MSNs, reaching statistical significance in late cultures (sEPSC frequency: DIV 40–65 *p* > 0.05, DIV 70–75 *p* = 0.0481 (Fig. 3G, J). However, there was no difference in sEPSC amplitude among detected sEPSCs between different culture conditions (Fig. 3H). These results imply that our microcircuit setup enables cortical afferents to make functional synapses with MSNs and enhances MSN excitatory synaptic activity.

Dendritic spines are the principal sites of synaptic integration, and their architecture is highly dynamic, reflecting the complex synaptic communication between neurons. Knowing that glutamatergic input profoundly modulates dendritic morphology of MSNs^31^, we examined whether we could observe a modified phenotype in dendritic spines in the presence of cortical glutamatergic innervation. We found that cortical excitatory afferents promoted early dendritic spine formation in MSNs which was detectable from DIV 40 (Fig. 3K, L). On the contrary, dendritic spines were virtually absent in early cultures of MSNs devoid of cortical projections, but emerged gradually over time and attained a similar density as in CN^+^MSN from DIV 55–60 onwards despite CN^-^MSN remaining relatively synaptically more quiescent (DIV 40–45: *p* = 0.0095; DIV 55–60: *p* = 0.0816, DIV 70–75: *p* > 0.999 CN^−^MSN vs. CN^+^MSN) (Figs. 3K and 2D). These results suggested the importance of cortical afferents in facilitating synaptic maturation of MSNs by prompting not only the formation but also the maturation of dendritic spines of striatal neurons, indicating the utility of our microcircuit in mimicking functional significance of the network connectivity in an in vitro model.

### Dopaminergic signalling promoted excitability maturation in a time-sensitive manner

**Fig. 4:**
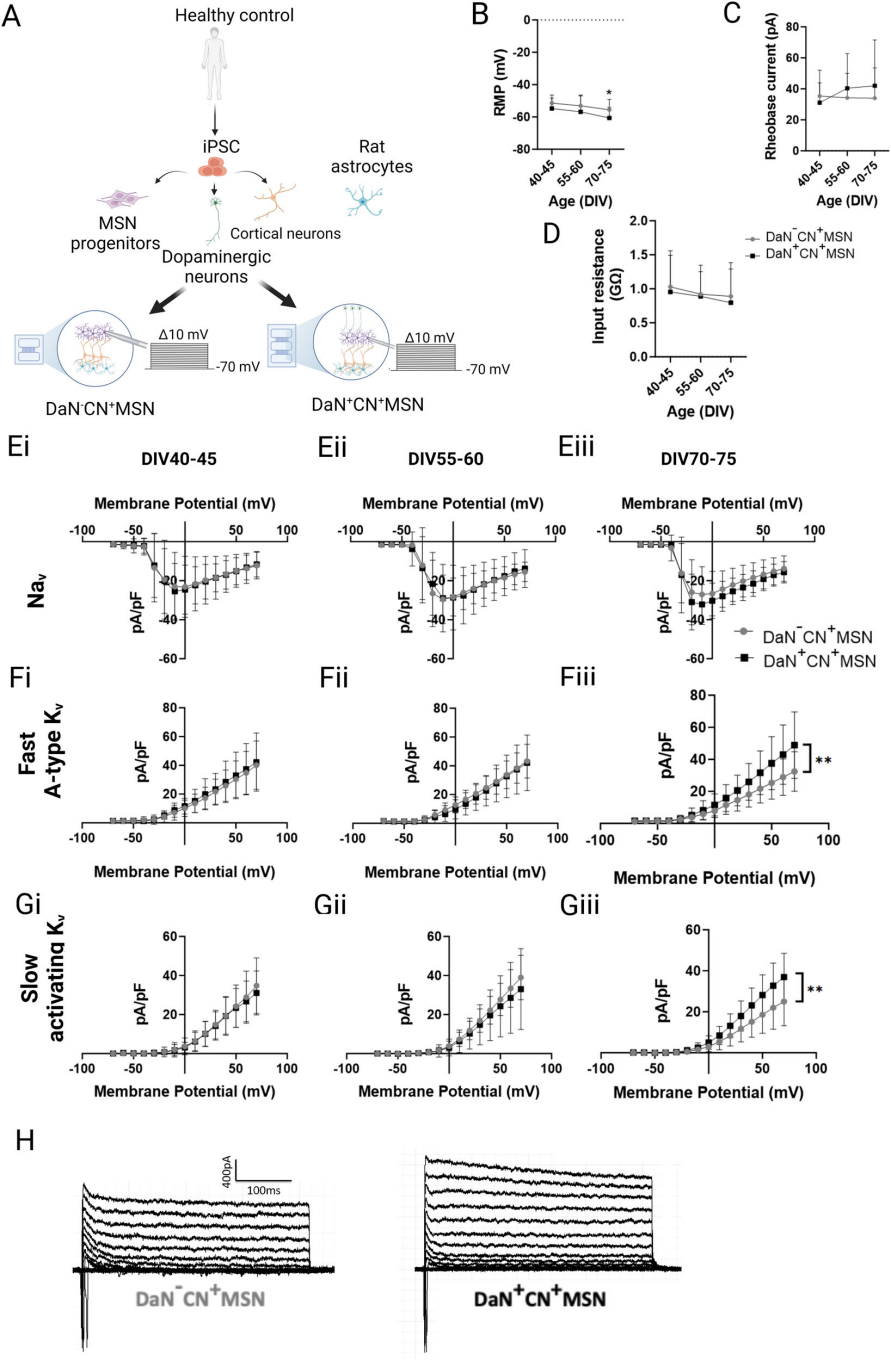
iPSC-DaNs enhanced the intrinsic maturation of iPSC-MSNs during maturation via maintenance of high potassium ionic conductance. **A** Schematics illustrating the culturing setup and whole cell patch-clamp recording of corticostriatal neurons in the presence or absence of DaNs, i.e., DaN^−^CN^+^MSN and DaN^+^CN^+^MSN, respectively, on microfluidic devices. Changes of passive properties including (**B**) Resting membrane potential (RMP), (**C**) rheobase current, and (**D**) input resistance over time, *n* = 9–35 recording MSNs per condition. Current density curves of (**E**) voltage-gated sodium channels, (**F**) fast A-type voltage-gated potassium, and (**G**) slow activating voltage-gated potassium channels over time, *n* = 19–30 recording MSNs per condition. **H** Representative fast A-type and slow activating K_v_ currents of DaN^−^CN^+^MSN and DaN^+^CN^+^MSN at DIV 70–75. All data are presented as mean ± SD, *N* = 2 differentiation experiments of 3 iPSC lines, 2-way ANOVA with Bonferroni post-hoc test, *p < 0.05, **p < 0.001.

To elucidate the direct effect of dopaminergic projections onto corticostriatal neurons, comparisons were made between CN^+^MSN (i.e., DaN^-^CN^+^MSN) and those circuited with DaNs (i.e., DaN^+^CN^+^MSN, Fig. 4A). We found that RMP of corticostriatal neurons became significantly more hyperpolarised with the long-term presence of DaN projections (DIV 40–45 DaN^+^CN^+^MSN vs DIV 70–75 DaN^+^CN^+^MSN *p* = 0.016), resulting in a significantly more hyperpolarised RMP of DaN^+^CN^+^MSN in late, but not early, cultures (DaN^-^CN^+^MSN RMP vs. DaN^+^CN^+^MSN RMP: DIV 40–45 *p* = 0.216, DIV 55–60 *p* = 0.222, DIV 70–75 *p* = 0.028) (Fig. 4B). This time-dependent hyperpolarisation of DaN^+^CN^+^MSN RMP was, however, not coupled with differences in rheobase current and input resistance of MSNs receiving dopaminergic inputs (Rheobase current: culturing condition *p* = 0.34; input resistance: culturing condition *p* = 0.38) (Fig. 4C, D). Observing the trends toward reduced excitability in the presence of dopaminergic afferents, we investigated the constituent voltage-gated currents of sodium (Na_v_) and potassium (K_v_) to delineate specific ionic channels responsible for the subtle differences. Coordinated activity of both Na_v_ and K_v_ plays the fundamental role in regulating neuronal excitability. The inward flux of sodium ions depolarises the cell membrane towards action potential threshold and triggers spontaneous firing while outward currents generated K_v_ underly resting membrane potentials and action potential after hyperpolarisation^32,33^. We found an increase in both the fast A-type and slow activating K_v_ in late cultures at DIV 70–75 (DaN^-^CN^+^MSN vs DaN^+^CN^+^MSN fast A-type K_v_ : DIV 40–45 *p* = 0.508, DIV 55–60 *p* = 0.391, DIV 70–75 *p* = 0.0061; DaN^-^CN^+^MSN vs DaN^+^CN^+^MSN slow activating K_v_ : DIV 40–45 *p* = 0.67, DIV 55–60 *p* = 0.270, DIV 70–75 *p* = 0.0015) (Fig. 4F–H) but no significant temporal changes in Na_v_ currents (DaN^−^CN^+^MSN vs DaN^+^CN^+^MSN: DIV 40–45 *p* = 0.927, DIV 55–60 *p* = 0855, DIV 70–75 *p* = 0.203) (Fig. 4E), in agreement with the more hyperpolarised RMP but unchanged rheobase current. The difference in K_v_ can result from either increased K_v_ of DaN^+^CN^+^MSN, or decreased K_v_ of DaN^−^CN^+^MSN, or both. When examining each condition independently over the same period, we observed a decrease in K_v_ conductance among DaN^-^CN^+^MSN (Supplementary Fig. 9A) but no changes among DaN^+^CN^+^MSN (Supplementary Fig. 9B). RT-qPCR of DaN^+^CN^+^MSN lysates over time confirmed the statistically stable expression between DIV 40 and 70 (all *p* > 0.05) of transcripts of most potassium channels, including voltage-gated potassium channels (Kv1.2, Kv4.2) as well as of inwardly rectifying potassium channels (Kir2.1 and Kir2.2) (Supplementary Fig. 10), all of which have been found to be among the principal contributors for potassium conductance and excitability of MSNs^21,34–37^. These data suggest that MSN excitability matures in DA-independent (early) and DA-dependent (late) stages of which the latter is likely to be regulated via maintenance of extensive K_v_ conductance.

### Early transient electrophysiological deficits of corticostriatal neurons circuited with *GBA-N370S* iPSC-DaNs

**Fig. 5:**
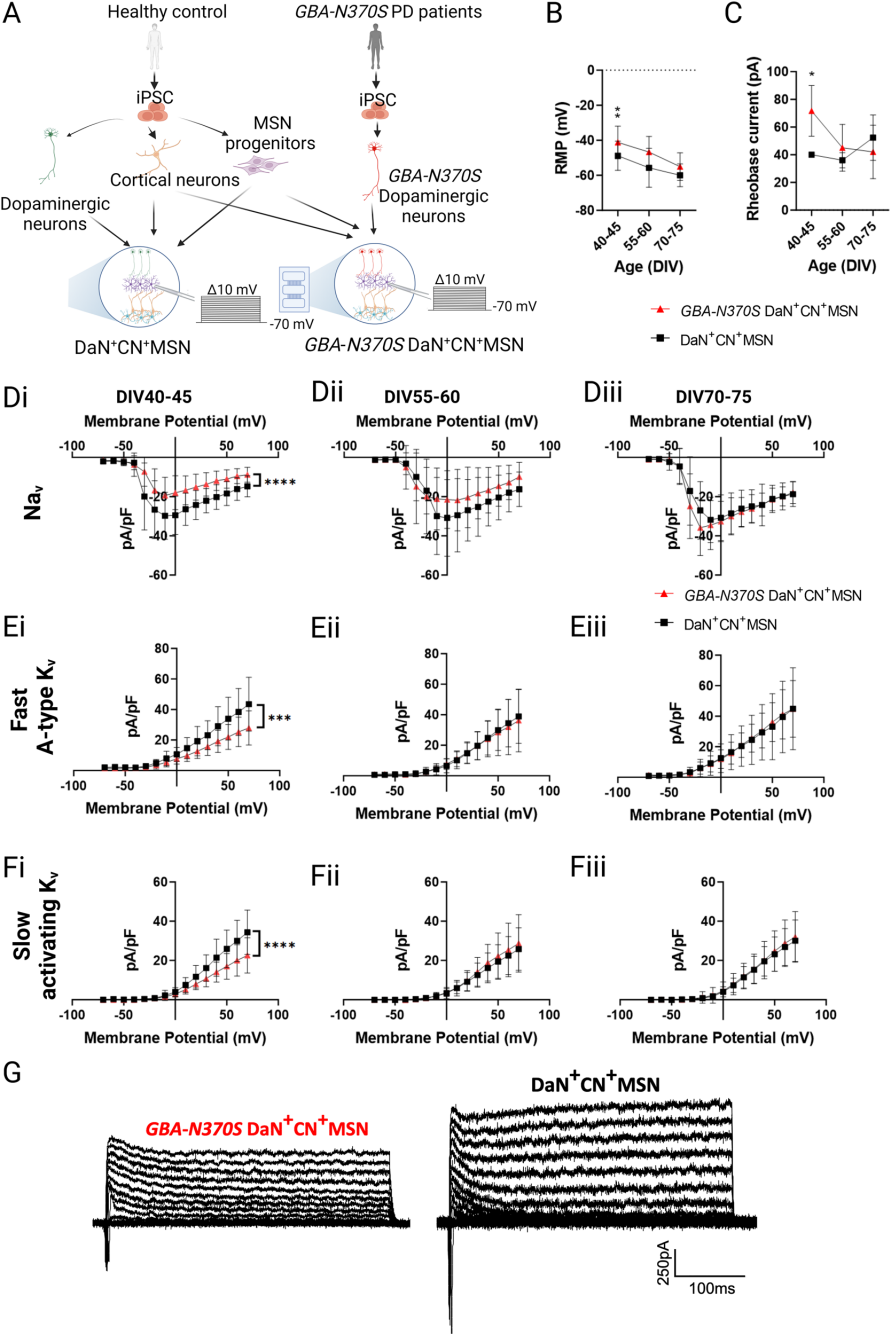
Early but transient current deficits and altered excitability of iPSC-MSNs circuited with *GBA-N370S* iPSC-DaNs. **A** Schematics illustrating the culturing setup and whole cell patch-clamp recording of DaN^+^CN^+^MSN and *GBA*-*N370S* DaN^+^CN^+^MSN. **B** Resting membrane potential (RMP), and (**C**) rheobase current of DaN^+^CN^+^MSN and *GBA*-*N370S* DaN^+^CN^+^MSN over time, *n* = 5–38 recording MSNs per condition. Current density curves of (**D**) voltage-gated sodium channels, (**E**) fast A-type voltage-gated potassium, and (**F**) slow activating voltage-gated potassium channels over time. **G** Representative differential fast A-type and slow activating K_v_ currents of DaN^+^CN^+^MSN and *GBA*-*N370S* DaN^+^CN^+^MSN at DIV 40–45. All data are presented as mean ± SD, *N* = 2–3 differentiation experiments of 3 iPSC lines, *n* = 27–32 recording MSNs at DIV 40–45, *n* = 14–16 recording MSNs per condition at DIV 55–60 and DIV 70–75. 2-way ANOVA with Bonferroni post-hoc test, ns *p* > 0.05, *p < 0.05, **p < 0.01, ***p < 0.001, ****p < 0.0001.

To dissect the impact of *GBA-N370S* iPSC-DaNs on the physiology of corticostriatal output neurons, we established the striatal microcircuit using iPSC-CNs and -MSNs from healthy controls connected with iPSC-DaNs from either healthy controls or PD patients carrying the *GBA-N370S* mutation (Fig. 5A). We compared the electrophysiological properties between corticostriatal neurons in microcircuit with either healthy iPSC-DaNs (DaN^+^CN^+^MSN) or *GBA-N370S* iPSC-DaNs (*GBA-N370S* DaN^+^CN^+^MSN). We found that while both groups exhibited increasingly hyperpolarised RMP over time, the RMP of DaN^+^CN^+^MSN was always more hyperpolarised than that of *GBA-N370S* DaN^+^CN^+^MSN, particularly statistically pronounced in early culture (DIV 40–45: *p* = 0.0033, DIV 55–60 and 70–75 *p* > 0.05) (Fig. 5B). A significant genotype effect was also observed for rheobase current at DIV 40–45, but not in later cultures (DIV 40–45: *p* = 0.0249, DIV 55–60 and 70–75 *p* > 0.05) (Fig. 5C).

We next explored whether the altered intrinsic excitability was coupled with relevant ionic channels. Indeed, we observed a substantial reduction in K_v_ among the *GBA-N370S* DaN^+^CN^+^MSN (DIV 40–45: slow activating K_v,_ DaN genotype *p* < 0.0001; fast A-type K_v_, DaN genotype *p* = 0.0001) (Fig. 5Di, Ei, Fi, G). Sodium ionic conductance of the *GBA-N370S* DaN^+^CN^+^MSN was also significantly decreased (DIV 40–45 Na_v_ *p* > 0.05), an effect seemingly contradicting the observation of reduced ionic potassium conductance and more depolarised RMP but potentially correlated with their increased rheobase. Recordings of MSNs from intermediate and late cultures between DIV 55–70 revealed resolution of genotypic effects previously observed at DIV 40–45 (Fig. 5D–F). Interestingly, there was no statistical difference in the expression of FOXA2-positive as well as FOXA2 and TH co-expressing cells between healthy control and *GBA-N370S* iPSC-DaN cultures (Supplementary Fig. 11), suggesting that the observed striatal electrophysiological phenotypes are independent of the abundance of TH-expressing neurons. Neither did we observe significant discrepancy in transcript abundance of key potassium channels, both voltage-gated and inwardly rectifying channels, expressed by MSNs cultured in microcircuit with either healthy control-derived or *GBA-N370s* iPSC-DaNs (Supplementary Fig. 12). Taken together, these data suggest an early role of the *GBA-N370S* mutation in iPSC-DaNs in altering electrophysiological properties of postsynaptic iPSC-MSNs independent of overt degeneration of dopaminergic neurons.

### PKA antagonism resolved early and transient ionic current deficits in corticostriatal neurons circuited with *GBA-N370S* iPSC-DaNs

**Fig. 6:**
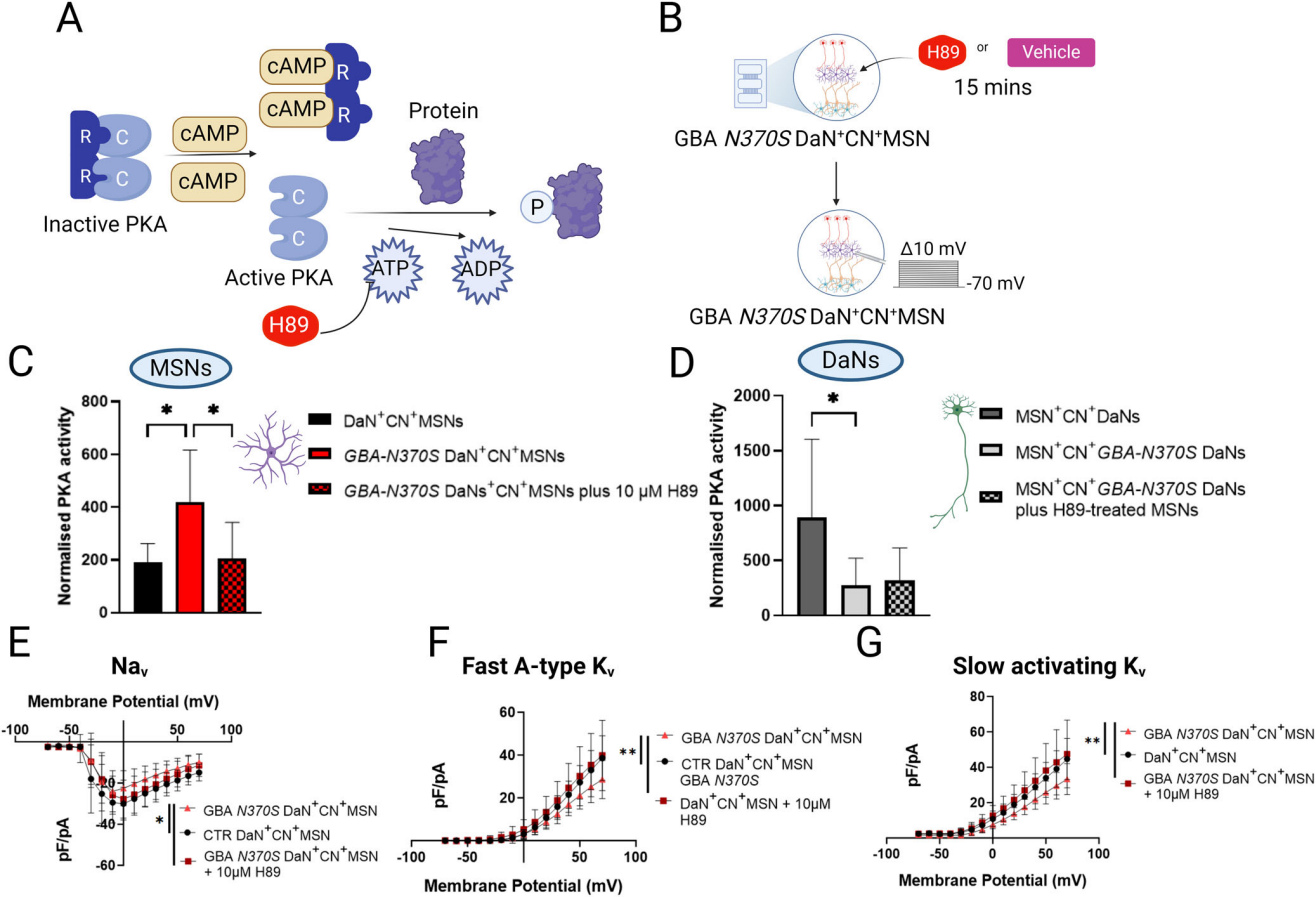
PKA antagonist rescued early current deficits of iPSC-MSNs circuited with *GBA-N370S* iPSC-DaNs. **A** Schematic demonstrating antagonistic mechanism of H89 in PKA-mediated phosphorylation signalling cascade. **B** Schematic illustrating treatment of PKA antagonist H89 on MSNs in the striatal presynaptic dyad; C = catalytic subunit, R = regulatory subunit, cAMP = Cyclic adenosine monophosphate, ATP = Adenosine triphosphate, ADP = Adenosine diphosphate. Quantitative measure of PKA activity of (**C**) MSNs in different culturing conditions including DaN^+^CN^+^MSN, *GBA-N370S* DaN^+^CN^+^MSN and *GBA-N370S* DaN^+^CN^+^MSN treated with PKA antagonist H89, Kruskal-Wallis test with Dunn’s correction test, and (**D**) DaNs in the corresponding cultures i.e., healthy DaNs, mutant *GBA-N370S* DaNs synapsed with healthy untreated MSNs or mutant *GBA-N370S* DaNs synapsed with H89-treated MSNs in their corresponding devices; Friedman test with Dunn’s correction test. *N* = 2 differentiations of 3 iPSC lines each genetic condition, *n* = 3 technical replicates; All data are presented as mean ± sem. Current density curves of (**E**) Na_v_, (**F**) fast A-type K_v_, and (**G**) slowly activating K_v_ of DIV 40–45 MSNs. Data are presented as mean ± SD, *N* = 2 differentiation experiments of 3 iPSC lines per a genetic condition, *n* = 17–22 recording MSNs per condition. 2-way ANOVA with Bonferroni post-hoc test unless otherwise stated, ns *p* > 0.05, *p < 0.05, **p < 0.01, ***p < 0.001.

The functional activity of many cellular channels, including voltage-gated sodium and potassium channels, is robustly regulated by PKA-mediated signalling pathway in response to fluctuations of external stimuli^38,39^. This mechanism has also been thought to be largely responsible for dopaminergic modulation from the midbrain on MSN excitability^40^. In MSNs both K_v_4.2 and K_v_1.2, the principal channel contributing towards fast A-type and slowly inactivating potassium current, respectively^34,35^, as well as Na_v_1.2 towards sodium current^41^, are negatively modulated by PKA-dependent phosphorylation of their subunits^42–44^. To examine whether PKA activity is involved in the early non-autonomous deficits of corticostriatal neurons affected by *GBA-N370S* iPSC-DaNs, we applied the cell-permeable PKA inhibitor H89 directly to iPSC-MSNs in the striatal chambers of the striatal microcircuit and performed whole-cell patch clamping of iPSC-MSNs at DIV 40–45 (Fig. 6A, B). PKA activity assay data suggested a doubling of PKA activity in iPSC-MSNs circuited with *GBA-N370S* iPSC-DaNs, as compared to those with healthy control-derived iPSC-DaNs (DaN^+^CN^+^MSN vs *GBA-N370S* DaN^+^CN^+^MSN *p* = 0.037) (Fig. 6C). The increase in PKA activity in the iPSC-MSNs was then reduced to the control level upon administration of 10 µM H89 to iPSC-MSNs for 15 min (DaN^+^CN^+^MSN vs *GBA-N370S* DaN^+^CN^+^MSN + 10 µM H89 *p* > 0.99, *GBA-N370S* DaN^+^CN^+^MSN vs *GBA-N370S* DaN^+^CN^+^MSN + 10 µM H89 *p* = 0.043) (Fig. 6C). These data demonstrated the phenotypic increase in PKA activity in MSNs synapsed with *GBA-N370S* iPSC-DaNs and the ability of H89 to normalise striatal PKA activity in vitro. DaN lysates were collected from the DaN chambers from the same devices as the healthy control-derived striatal lysates, and a significant reduction in PKA activity was seen in *GBA-N370S* iPSC-DaNs (i.e., MSN^+^CN^+^*GBA-N370S* DaN) relative to healthy control-derived DaNs (i.e., MSN^+^CN^+^DaN) (MSN^+^CN^+^DaN vs MSN^+^CN^+^*GBA-N370S* DaN *p* = 0.044) (Fig. 6D). This deficit in PKA activity in *GBA-N370S* iPSC-DaNs remained in the mutant *GBA-N370S* iPSC-DaNs synapsed with iPSC-MSNs treated with H89 (*p* > 0.05), confirming the fluidic isolation of different neuronal compartments. Treatment of iPSC-MSNs with H89 could correct the genetic effect of *GBA-N370S* iPSC-DaNs on iPSC-MSNs Na_v_ and K_v_ current density at DIV 40–45 to healthy control levels (Fig. 6E–G), suggesting the involvement of potential PKA-associated mechanism in mediating excitability of corticostriatal neurons via ionic conductance.

## Discussion

The present study represents the first attempt at recapitulating an in vitro neuronal circuit model of the human striatal synaptic dyad using an open chamber-based microfluidic platform to reveal the physiological importance of connectivity-driven inputs on human striatal functions. Our data suggest an early nonautonomous striatal electrical dysfunction in MSNs in the presence of the *GBA-N370S* mutation within presynaptic DaNs that was associated with increased striatal PKA activity. Interestingly, in this model MSNs showed dynamic cell-autonomous PKA-dependent modulation of ionic channel physiology as illustrated by the resolution of early striatal deficits by specific antagonism of striatal PKA activity, despite the persistent presence of mutant DaNs.

Previous strategies to investigate human striatal neuron (patho)physiology remain largely limited in monocultures of a single neuronal type^18^, with more recent successes in developing a neuronal network architecture^9,10^, highlighting the necessity of more sophisticated cellular models that can better capture the surrounding neuronal connections critical for the emergence of functional characteristics of neurons. Indeed, although our MSNs in monoculture displayed a temporal marker expression profile reminiscent of the developmental trajectory reported from the Human Brain Transcriptome (https://hbatlas.org), combined cortical glutamatergic and dopaminergic inputs further facilitated functional maturation of the MSNs beyond the benefits of long-term culturing. Our results corroborated the relevance of cortical glutamate in promoting excitatory synaptic maturity of MSNs^45^ as well as the time-sensitive influence of dopaminergic transmission on MSNs excitability maturation, likely through modulation of potassium channels, previously reported in rodent models^21^. It should also be noted that the glutamatergic input onto MSNs originates from not only the cortex but also the thalamus which makes up ∼40% of glutamatergic synapses on MSN dendrites and converges with corticostriatal afferents indiscriminately onto both MSN subtypes^46^.

A significant highlight of this open microfluidic-based circuit approach is its customisability. The modular nature of this setup allows great flexibility for reconstructing the compartmentalisation of different neuronal populations and the directionality of their specific connectivity. Hence, virtually any microcircuit can be established applying a similar design concept. Targeted manipulation to specific compartments is also permitted with great ease as exemplified by our striatal PKA treatment paradigm. Immunostaining and electrophysiological results indicate that our approach has captured the oriented connectivity resembling that in vivo and that functional glutamate and dopamine transmission exists.

Most DA reported effects on MSN intrinsic excitability involve modulation of ionic potassium and sodium channels. Considering that the ATP competitive analogue H89 produced similar amplification of both Na_v_ and K_v_ in our study, the observed elevation of striatal PKA activity likely occurred upstream of cAMP in the PKA signalling cascade, potentially through excessive Dopamine 1 receptor (D1R) activation followed by stimulation of adenylyl cyclase-cAMP cascade^40^. D1R antagonist has been shown previously to decrease PKA activity in direct pathway MSNs^47^. Voltage clamp work has confirmed that D1R stimulation reduced Na_v_ currents by limiting channel availability^48^ while lessening K_v_ peak current amplitude through altering channel inactivation kinetics^43^. Furthermore, D1R agonist was found to reversibly reproduce the reduction in Na_v_ and K_v_ current amplitude, similarly through PKA-mediated phosphorylation of channel subunits, and improved striatal firing probability^49,50^. Considering that the majority of our culture was composed of direct pathway MSNs (dMSNs) expressing mainly pro-dynorphin (Supplementary Fig. 3), it is reasonable to hypothesise the potential role of D1R in our observed effect. Additionally, given that the striatal excitability in the absence of dopaminergic projections at DIV 40–45 (Fig. 4Ei, Fi, Gi) did not phenocopy the early striatal deficit synapsed with *GBA-N370S* iPSC-DaNs at the same timepoint (Fig. 5Di, Ei, Fi), we speculate that increased, rather than reduced, DA content at the nigrostriatal synapses is a more logical explanation. This hypothesis aligns with previous work on other models of PD-related familial mutations reporting early cell-autonomous hyperexcitability of midbrain DaNs prior to overt neurodegeneration^51–53^ which could result in increased PKA activity in dMSNs^47^, but contrasts with a single report on *GBA-N370S*-associated reduced spontaneous firing and basal DA release by Woodard and colleagues^54^ in monocultured DaNs.

Our mix-and-match cultures of healthy iPSC-MSNs and -CNs with *GBA-N370S iPSC-*DaNs allow us to understand the effect of mutant DaNs on MSNs and the associated role in the progression of PD among *GBA1* mutation carriers, a component that would have been masked if investigating only all-GBA cortico-striato-nigral cultures^11^. Our data suggest early changes in striatal function which are absent at later timepoints, which in turn suggests a compensation effect. This could likely be intrinsic to non-mutant MSNs, and so we propose that *GBA1* mutation-driven deficits in DaNs emerge early but in themselves are insufficient to induce long-term dysfunction in the motor circuit regulated by the basal ganglia. This evidence of a potentially compensative capacity could potentially explain the low percentage of *GBA1* mutation carriers eventually developing PD. It is, therefore, interesting to speculate that MSNs might be able to compensate for PD disease-driving deficits elicited by DaNs, thereby masking initial disease stages and the prodrome. In fact, altered functional connectivity of the nigrostriatal and striatocortical circuits have been reported in asymptomatic carriers of PD-related mutations^55^, and linked to prodromal non-motor features such as rapid eye movement sleep behaviour disorder (RBD)^56^. While major therapeutic efforts have been channelled towards reversing DaN pathology, our results suggest that the rescue of the striatal deficits is an attractive alternative to preserve the integrity of the basal ganglia output. If compensation is indeed functionally implicated in genetic disease as our data suggest, then therapies targeting MSNs might be able to provide patients with symptomatic relief. Future studies could mimic the full genetic landscape of *GBA-N370S* carriers by deriving all three neuronal populations of the microcircuit from the same *GBA-N370S* iPSC lines. Pairwise comparisons of striatal function from microcircuits in which the *GBA1* mutation burden is carried by different, all, or none of the component cell types mixed with healthy controls could delineate contributing pathophysiological roles of different neuronal populations of the striatal presynaptic circuitry to the net debilitating burden of the *GBA1* mutation and guide therapeutic efforts.

We have demonstrated a PKA activity-related mechanism by which mutant *GBA-N370S* DaNs induced early changes in healthy control-derived iPSC-MSNs preceding neurodegeneration. We also observed a reduction in PKA activity in *GBA-N370S* iPSC-DaNs relative to healthy control-derived DaNs (Fig. 6D) which is consistent with the previously reported downregulation of PKRACB transcript expression from DaN monocultures harbouring the same genetic burden^14^. Mutation of the PKA regulatory subunit *PRKAR1B* has also been reported in a novel familial neurodegenerative condition with parkinsonism and dementia^57^, and *Prkar1b*-knockout rats exhibit body tremor^58^. Moreover, reduced PKA activity in DaNs has been implicated in PD pathophysiology in other PD-related rodent mutation models such as *LRRK2* R1444C/G/H^59,60^ and *PINK1*^61^. Likewise, increased PKA activity was thought to be protective against PD^47,62^. However, the exact mechanistic link between *GBA1* mutations and PKA activity in nigral neurons remains to be further investigated. Nevertheless, it is well documented that the large network of lysosome genes including *GBA1* is moderated by a master regulator transcription factor EB (TFEB)^63^ which in turn is the downstream substrate of PKA-mediated phosphorylation via mTORC1^64^. Considering our previous observation indicating dysregulation of lysosomal/autophagic signalling in monocultured DaNs carrying *GBA-N370S*^12,14^, we hypothesize that multiple cellular perturbations in nigral DaNs burdened by the *GBA-N370S* mutation might be initiated by reduced activity of the master kinase PKA which disrupts various associated neuronal functions potentially via lysosomes and/or lysosomal-related organelles. In addition, PKA activation is closely related to proteasomal activation^65,66^, which is a necessary function often compromised in PD^67,68^. It is also interesting to speculate that reduced PKA activity could alter protein homoeostasis through reduced proteasomal function, and so, in turn, cause cellular dysfunction. From a preferential vulnerability point of view, protected cells like MSNs might be able to find compensation mechanisms and recover from these effects, while vulnerable cells such as DaNs may not be able to^69^.

PKA was thought to positively modulate neurotransmitter release in most synapses via a combination of factors including phosphorylation of various proteins involved in synaptic vesicle exocytosis^70–72^. In vivo studies have suggested PKA phosphorylation-mediated increase in DA release via activation of TH enzymatic activity^73,74^. The involvement of PKA in neurotransmitter release suggests that mutant *GBA-N370S* may affect DA striatal content, and that the pathophysiology of *GBA-N370S* variant-mediated PD may result from a disruption of the tight modulation of PKA activity.

In conclusion, we have described an open microfluidic-based neuronal microcircuit model which has used iPSC-derived neurons to recapitulate the presynaptic network relevant to in vivo striatal neurons in vitro. This approach allowed us to explore electrophysiological features of human MSNs over time in both the healthy and diseased state. We envision greater use of this modular circuit-based model in addressing sophisticated functional questions of human physiology and pathology.

## Methods

### Acquisition and maintenance of iPSC lines

iPSC lines used in this study were derived from human skin biopsy fibroblast acquired with informed consent and ethical approval (Ethics committee: National Health Service, Health Research Authority, NRES Committee South Central, Berkshire, UK, REC 10/H0505/71). The iPSC lines have been previously characterized: 3 control iPSC lines SFC067-03-01 (RRID:CVCL_RD75), SFC156-03-01 (EBiSC Cat# STBCi101-A, RRID:CVCL_RD71)^14^, and SFC856-03-04 (RRID:CVCL_RC81), 3 *GBA*-*N370S* PD lines MK082-26 (RRID:CVCL_IJ04)^14^; MK088-01 (EBiSC Cat# UOXFi003-A), and MK071-03 (EBiSC Cat# UOXFi001-B)^12^ (Table 1). All iPSCs were maintained on Matrigel™ (Corning)-coated plates under feeder-free culture condition with mTSER1™ (StemCell Technology) supplemented with 1% penicillin/streptomycin (P/S) (Life Technologies). Medium was changed daily, and cultures were passaged 1:2-3 using TrypLE Express™ (Life Technologies) when they reached 90-100% confluency in the presence of Rock inhibitor (Bio-Techne). Every new batch of expansion of an iPSC clone was karyotyped and SNP tested to assess genetic integrity prior to further use. Batches of cultures was tested for mycoplasma (Mycoalert, Lonza). iPSCs were expanded for maximum 2 weeks prior to differentiation at passages between 15–30.

**Table 1:** Demographic details of iPSC lines used in this study

Genotype	Cell Line	Clone	Sex (F/M)	Age
Healthy controls	SFC067-03	1	M	72
	SFC156-03	1	M	75
	SFC856-03	4	F	78
*GBA-N370S*	MK071	3	F	81
	MK082	26	M	51
	MK088	1	M	46

### Generation of iPSC-derived MSNs

3 iPSC control lines (SFC067-03-01, SFC156-03-01, and SFC856-03-04) were differentiated into MSNs using condition modified from previously established protocols^7,8^. In brief, iPSCs were plated at 75,000 cells/cm^2^ and maintained until 85% confluence. Culture was switched to neural induction medium containing DMEM/F12 (Life Technologies), 1% MEM Non-Essential Amino Acids (NEAA), 1% Glutamax, 2% B27 without vitamin A (all from ThermoFisher), 1% P/S, 200 nM LDN193189 (Sigma), 10 μM SB431542 (Abcam) and 4 μM XAV (Tocris). On DIV 4, culture was pre-incubated with ROCK Inhibitor (Y-27632) for 1 h before being passaged in 1:2 ratio. Culture was passaged in 1:2 on DIV 8 and switched to differentiation medium containing DMEM/F12, 1% P/S, 1% NEAA, 2 mM L-glutamine (Life Technologies), 2% B27, 100 nM LDN193189, and 4 μM XAV. Activin A was added to from DIV 12 till DIV 23.

On day 16, the culture was either cryopreserved for future experiments or replated at 65,000 cells/cm^2^ onto freshly coated plates or coverslips. Coating included 10 and 100 μg/ml Poly-D-Lysine (Sigma-Aldrich) overnight for plastic plates and glass coverslips, respectively, followed by 1:100 Matrigel™ for 1 h. Neuronal progenitor cells were cultured in the maturation medium SynaptoJuice® as previously reported^8^. Cultures were treated with 200 nM AraC on DIV 18 to arrest proliferation of dividing non-neuronal cells capable of DNA synthesis. AraC concentration in the culture was gradually diluted with subsequent media changes.

### Generation of iPSC-derived CNs

Generation of CNs from 3 iPSC healthy control lines (SFC067-03-01, SFC156-03-0, and SFC856-03-04) was adapted from a previously detailed protocol^22^. In brief, neural development was induced with dual SMAD inhibitors 10 μM SB431542 (Tocris) and 100 nM LDN (Sigma). Following formation of a dense neuroepithelial sheet, the whole sheet was enzymatically lifted using Dispase (Life Technologies) and broken down into smaller clumps to encourage development of neural rosettes. Rosettes were maintained and expanded on laminin-coated wells till DIV 30 at which point cultures were either cryopreserved or dissociated into single cells for further differentiation. At DIV 30 cortical neurogenesis was induced by doxycyline-dependent overexpression of *NGN2* via co-transduction of LV-TetO-mNgn2-T2A-Puro and LV-Ubiq-rTA lentiviruses in the cultures. Transduced cells were incubated for 2 days before being selected with fresh media supplemented with 1 µg/ml puromycin for another 48 h prior to final replating.

### Generation of iPSC-derived DaNs

All 3 control and 3 *GBA N370S* PD iPSC lines were differentiated into DaNs as previously described^14^ with added modifications^75^ (see detailed Method and Supplementary Fig. 5). In brief, iPSCs were dissociated into single cells, plated on Geltrex-coated plastic plates at 125,000 cells/cm^2^ and cultured until confluence. iPSCs were then patterned toward floor-ventral midbrain precursors for 10 days using a KnockOut™ DMEM and KnockOut™ serum replacement (both from ThermoFisher)-based cell culture media supplemented with 10 μM SB431542 (Tocris) between DIV 0-5, 100 μM SHH-C24II (Biotechne), 100 ng/ml FGF8 (Peprotech), and 2 μM Purmophine (StemCell Technology) between DIV 1-7, and 3 μM CHIR99021 (Biotechne) DIV 3-10. Day 10 precursors were expanded with weekly 1:2-3 passaging for 20 days in DIV 10 media. Post-expansion DIV 29 precursors could be cryopreserved or immediately further differentiated and matured into post-mitotic midbrain neurons until DIV 38 for final replating onto microfluidic devices.

### Immunocytochemistry

Cultures were fixed with 2% PFA for 20 min at room temperature (RT), rinsed with PBS, and antigen retrieved in citrate buffer pH 6.0 (ThermoFisher) in water bath at 80 °C for 5 min. Samples were left to rest for 10 mins at room temperature before being permeabilised and blocked in PBS, 10% donkey serum and 0.01% of Triton X-100 for 10 min. Incubation of primary antibody in PBS and 10% donkey serum was performed overnight at 4 °C. Samples were washed with PBS and subsequently incubated in species-appropriate Alexa Fluor© secondary antibody in PBS with 10% donkey serum for 1 h at RT. The list of primary and secondary antibodies used for immunostaining is available on Zenodo (10.5281/zenodo.10650790). Images were acquired on the Opera Phenix High-content Screening system (PerkinElmer) or the Invitrogen EVOS ™ FL Auto (ThermoFisher) cell imaging system and subsequently processed on Harmony (Perkin Elmer; RRID:SCR_018809), ImageJ (RRID:SCR_003070) or CellProfiler (RRID:SCR_007358), respectively.

### Reverse transcription-qualitative polymerase chain reaction (RT-qPCR)

Total RNA of MSN cultures over time was extracted and purified from cell lysates using RNeasy Mini kit (Qiagen) according to manufacturer’s instructions. Extracted RNA was quantified and assessed with a Nanodrop (DS-11, Denovix). RNA was reverse transcribed into cDNA using a superscript III reverse transcriptase kit (Life Technologies). qPCR was performed with standard SYBR green (ThermoFisher) in StepOnePlus thermal cycler (Life Technologies). Full list of the primers used in this study are available on Zenodo (10.5281/zenodo.10650790).

### Microfluidic fabrication

A custom master mould containing both duo- and trio-chambers was manufactured by Microliquid (Spain). The microfluidic device was made from the mould by pouring a silicon elastomer SylGARD 184, Dow Corning © pre-mixed with its curing agent in the 1:10 ratio. Polymerisation occurred when incubating the PDMS-filled mould for 3 h at 60 °C. Final devices were manually cut out from the negative cast and washed with 100% ethanol. Cut-out devices were air-dried in tissue culture hood and subsequently placed on ethanol –sterilised 19 mm-diameter coverslips to form an instantaneously tight seal. Both the chambers and microchannel area were coated with poly-D-lysin (0.1 mg/ml) overnight followed by Geltrex™ (ThermoFisher) prior to cell culturing.

### Electrophysiology

Whole-cell recordings of MSNs on coverslips were performed at 27 °C in static bath with periodic change of external solution following every new recording cell. The external solution contained: 2.4 mM KCl, 167 mM NaCl, 10 mM Glucose, 10 mM HEPES, 1 mM MgCl_2,_ and 2 mM CaCl_2_ adjusted to 300 mOsmol/l and pH 7.36 with NaOH. Neurons were patched with a borosilicate glass pipette (8-12 MΩ) pulled using a Sutter P-97 Flaming Brown puller (Sutter Instrument Company) and filled with an internal solution consisting of: 140 mM C_6_H_11_KO_7,_ 6 mM NaCl, 1 mM EGTA, 4 mM MgATP, 0.4 mM Na_3_GTP, 10 mM HEPES, and 0.01% Neurobiotin (SP-1120) adjusted to 290 mOsmol/l and pH 7.3 using KOH. Data were acquired with MultiClamp 700B amplifier (Molecular Devices) and Digidata 1550B digitiser (Molecular Devices) and analysed in Clampfit 10.7 (Molecular Devices). Cells were always clamped at −70 mV unless otherwise stated. Access resistance was constantly monitored; cells with access resistance greater than 50 MΩ were excluded. All recordings were made within 2 h of coverslip preparation.

Cell capacitance and input resistance were automatically reported from Clampex membrane test sampled at 33 kHz. Resting membrane potential (RMP) was acquired immediately upon successful break-in in current clamp with zero current injection. Liquid junction potential was estimated at 12 mV using JPCal^76^ and adjusted for in RMP recordings.

Na_V_ and K_V_ currents were recorded in voltage clamp from the holding potential of −70 mV using a series of 400 ms square voltage steps of 10 mV increments from −70 mV to +70 mV. Signals were sampled at 10 kHz and filtered at 2 kHz. Leak subtraction was applied with 4 sub-sweeps and a settling time of 250 ms. Evoked action potentials were recorded in current clamp by injecting 500 ms current steps in 10 pA increments from −10 pA to +130 pA.

Spontaneous EPSC and IPSC were recorded in voltage-clamp mode with 20x gain at one another’s reversal potential i.e., −70 mV and 0 mV, respectively. The internal solution for spontaneous synaptic activity recordings was Cs-based containing 140 mM C_6_H_11_CsO_7,_ 6 mM NaCl, 1 mM EGTA, 4 mM MgATP, 0.4 mM Na_3_GTP, 10 mM HEPES, and 0.01% Neurobiotin adjusted to 290 mOsmol/l and pH 7.3 using CsOH. All sEPSC and IPSC recordings were 1–4 min in duration and low-pass Bessel 8-pole filtered post-hoc. Spontaneous postsynaptic events and event magnitude were automatically detected based on absolute magnitude difference from the baseline (>10 pA for sEPSCs and >15 pA for sIPSCs) using threshold search function on Clampfit (RRID:SCR_011323). Putative EPSC or IPSC events were excluded based on template-dependent criteria including rise time and half-width (<4 ms and <1.2 ms, respectively) and manually validated to reject false-positive events.

H89 (Tocris) was dissolved in dH2O and were media applied to the middle chamber to the final concentrations of 10 μM for 15 min prior to wash off and recording.

All patched neurons were post-hoc labelled with Streptavidin-Alexa Fluor 488 conjugate (ThermoFisher) and DARPP32 (Sigma) to ascertain the MSN identity. Only neurobiotinylated neurons that co-expressed DARPP32 were selected for analysis.

### Dendritic spine visualisation and quantification

Patched coverslips of MSNs were immunolabelled for DARPP32 (as described above) and mounted onto SlowFade ™ Diamond Antifade mountant (ThermoFisher). Detection and imaging of DARPP32-positive Neurobiotin™-filled neurons were captured under Olympus FluoView FV1000 confocal microscope with argon and solid-state laser with 488 nm and 559 nm excitation, respectively. Z-stacks of images were sampled sequentially at resolution of 1024 * 1024 pixels, with 60x oil-immersion objective (NA = 1.40), at 1.05 µm steps as optimised by Nyquist sampling theorem. Dendritic branches of biotinylated neurons were captured at 3x software zoom. Images were segmented, reconstructed and automatically rendered using filament and spine detection module in Imaris 9.6.0 (Bitplane, South Windsor, CT, USA, RRID:SCR_007370) which identified dendrites and detect protrusions along the dendritic filament length. Putative spines at branch points or disconnected dots were manually excluded.

### PKA activity assay

PKA activities were measured using the PKA Colorimetric Acitivity Kit (ThermoFisher) according to manufacturer’s guidelines. Protein concentration of the same samples was quantified using Pierce™BCA Protein Assay Kit (ThermoFisher) following manufacturer’s instructions. Both plates were read for absorbance in a PHERAstar® microplate reader (BMG Labtech). Protein concentration and PKA activity values were inferred from their respective standard curves. Final PKA activity read-outs were normalised against the sample protein concentrations.

### Neurotransmitter release assay (Glutamate)

For tonic glutamate release quantification, the media was first changed to prewarmed HBSS + + (with 2.4 mM KCl) and incubated for 10 min before collection. For evoked release quantification, prewarmed HBSS++ (with 40 mM KCl) was added to cells and incubated for 5 min. Supernatants were collected in both instances and stored in −20 °C until further use. Tonic and evoked level of glutamate release were measured using Glutamate Assay Kit (ab83389) according to the manufacture’s protocol booklet. Protein concentrations of the samples were quantified using Pierce™BCA Protein Assay Kit (ThermoFisher) following manufacturer’s instructions. The OD was read at 450 nm in a PHERAstar® microplate reader (BMG Labtech). Protein concentration and glutamate concentrations were determined from their respective standard curves.

### Neurotransmitter release assay (Dopamine)

Day 70 iPSC-DANs at 500,000 cells/well of a 24 well plate were washed once with phosphate buffered saline (PBS) and incubated in 100 µL of Ringer’s buffer (1.2 mM CaCl2, 148 mM NaCl, 0.85 mM MgCl2, 2.7 mM KCl or 40 mM KCl, pH 7.4, 300 Osmol) for 5 min. Supernatant was snap-frozen on dry ice and stored at −80 °C in perchloric acid (PCA). Prior to loading on HPLC column, samples were spun at 10000 g for 10 min. Mobile phase (13% HPLC grade methanol, 0.12 M Sodium phosphate monobasic dihydrate (NaH2PO4), 0.8 mM Ethylenediaminetetraacetic acid (EDTA) and 0.5 mM 1-Octanesulphonic acid sodium salt (OSA), pH 4.6) was run at flow rate of 1 mL/min. Samples were run on a 4.6 × 150 mm Microsorb C18 reverse-phase column and detected using Decade II ECD with a glassy carbon working electrode (Antec Layden) set at 0.7 V with respect to an Ag/AgCl reference electrode. Concentrations of monoamines were calculated compared to known standards.

### Statistical analysis

For biochemical analysis including RT-qPCR, immunohistochemical quantification, PKA activity assay, and neurotransmitter release assay, three technical replicates were used per cell line per differentiation per experiment and each data point represents average values of the triplicate of a single experiment. Hence, all biochemical data were presented as mean ± Standard error of the mean (sem) unless otherwise stated. For electrophysiological and spine density analysis, each data point represented a recording neuron, and therefore, these data were presented as mean ± standard deviation (SD). Raw data was tested for normality (Shapiro–Wilk normality test) and statistical comparison of the means was performed using unpaired Student’s *t*-test, two-way ANOVA with Bonferroni post-hoc test, or Kruskal–Walli’s test or Friedman test corrected with Dunn’s multiple comparison test when appropriate. Significant differences were considered at *p* < 0.05. All statistical analyses were performed on GraphPad Prism 10 (GraphPad Software, RRID:SCR_002798).

### Supplementary information

Supplementary material

Related Manuscript File

## Data Availability

The data that support the findings of this study are deposited on Zenodo (10.5281/zenodo.10650790). All details of the primers, antibodies, cell lines, and software used in this work are available on Zenodo (10.5281/zenodo.10650790). Protocols associated with this work can be found on protocols.io (10.17504/protocols.io.x54v9dw61g3e/v2).
