# Supplementary material for: Early deficits in an in vitro striatal microcircuit model carrying the Parkinson’s GBA-N370S mutation
Source: NPJ Parkinsons Dis. 2024 Apr 12;10:82. doi: 10.1038/s41531-024-00694-2 (PMC11014935; doi:10.1038/s41531-024-00694-2)
Supplement: Supplementary file 1 — Supplementary material [file 41531_2024_694_MOESM1_ESM.pdf]

Sup. Fig. 1

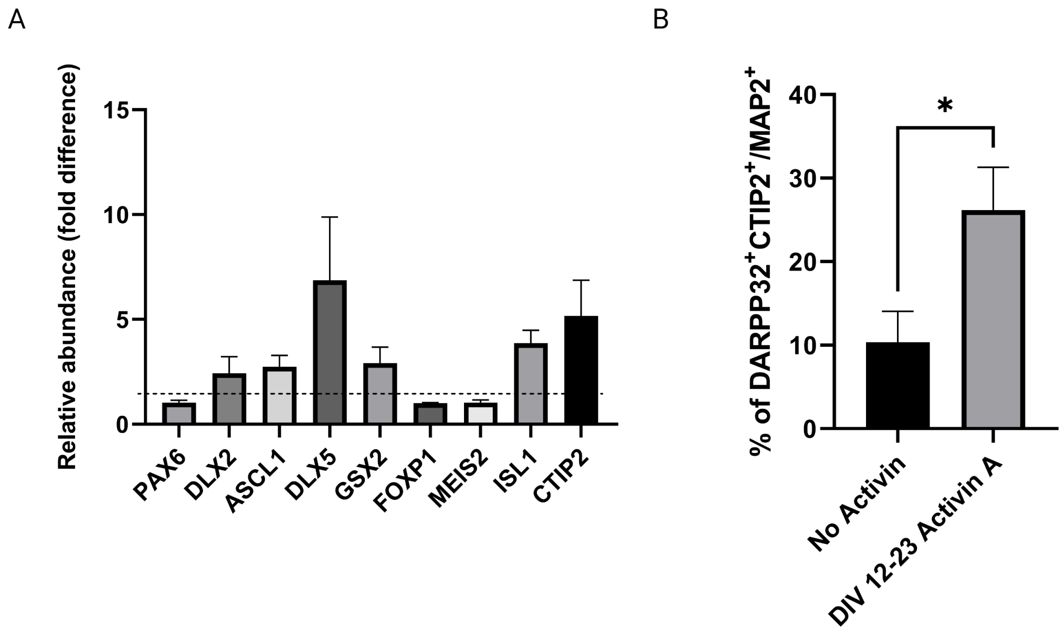

Supplementary Figure 1: Activin A promoted LGE-striatal development. (A) Relative transcript abundance of selected LGE and MSN progenitor markers at DIV 14 when cells were treated with Activin A at DIV 12 normalised against those that were not; dotted line represents fold difference of 1. (B) Percentages of post-mitotic neurons co-expressing DARPP32 and CTIP2 at DIV 40 in the presence or absence of Activin A from DIV 12-23. Mean ± sem are depicted on graphs. N = 2-3 differentiations, n = 3-4 iPSC lines, and 3 technical replicates, unpaired Student's t-test. * $p < 0.05$.

Sup. Fig. 2

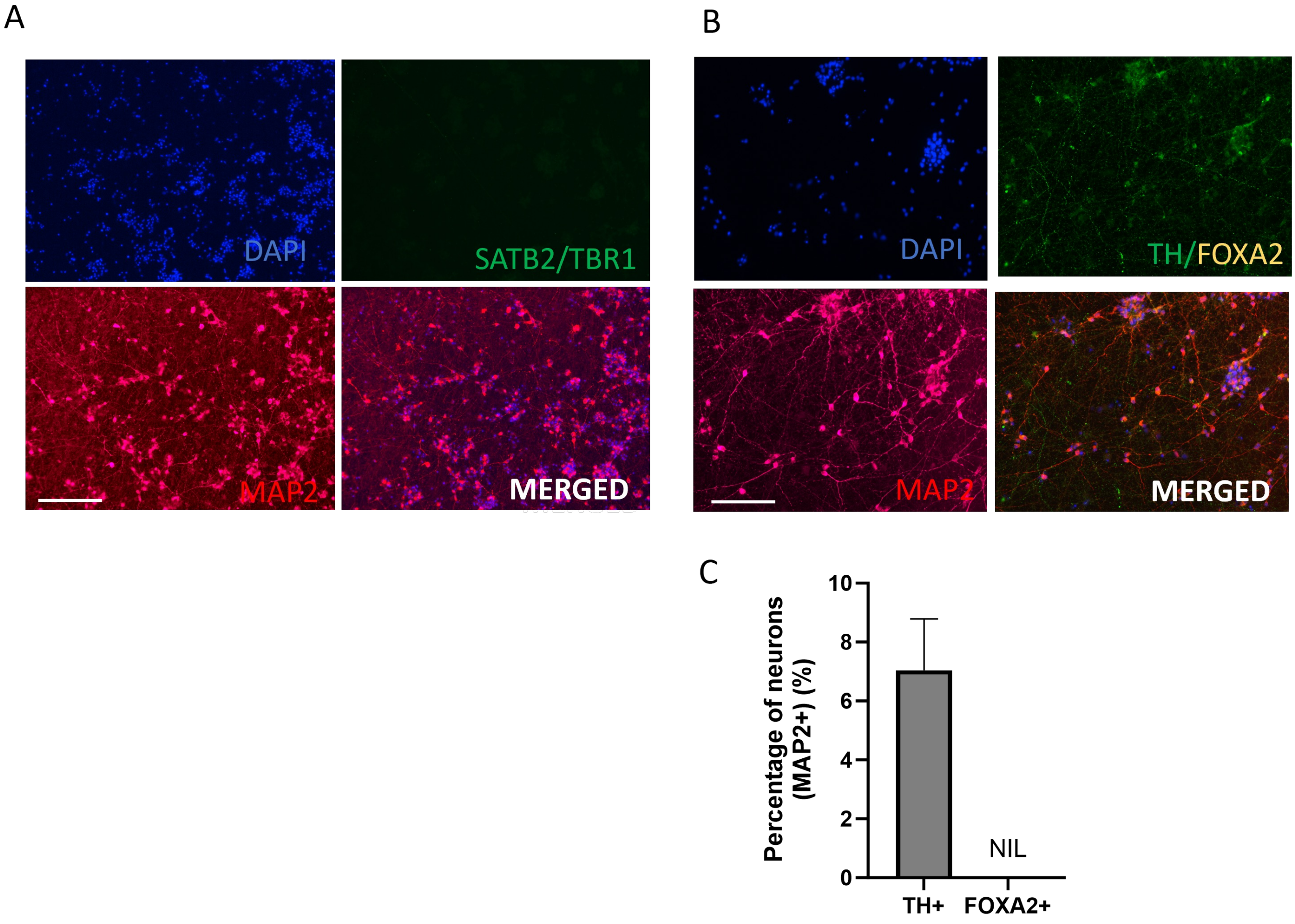

Supplementary Figure 2: Expression of non-MSN specific proteins. (A) Representative fluorescent immunocytochemical images of MSN cultures suggest no expression of cortical specific markers SATB2 or TBR1 (green). (B) Immunocytochemistry of MSN cultures confirmed presence of TH (green) -positive neurons which are not of floor-plate origin i.e. FOXA2 (yellow)-negative as well as TH-positive neurites. (C) Percentage of neurons expressing either TH or FOXA2 in iPSC-MSN cultures. Data are presented as mean ± sem, of 3 healthy control iPSC lines. Scale bar = 100 µm.

Sup. Fig. 3

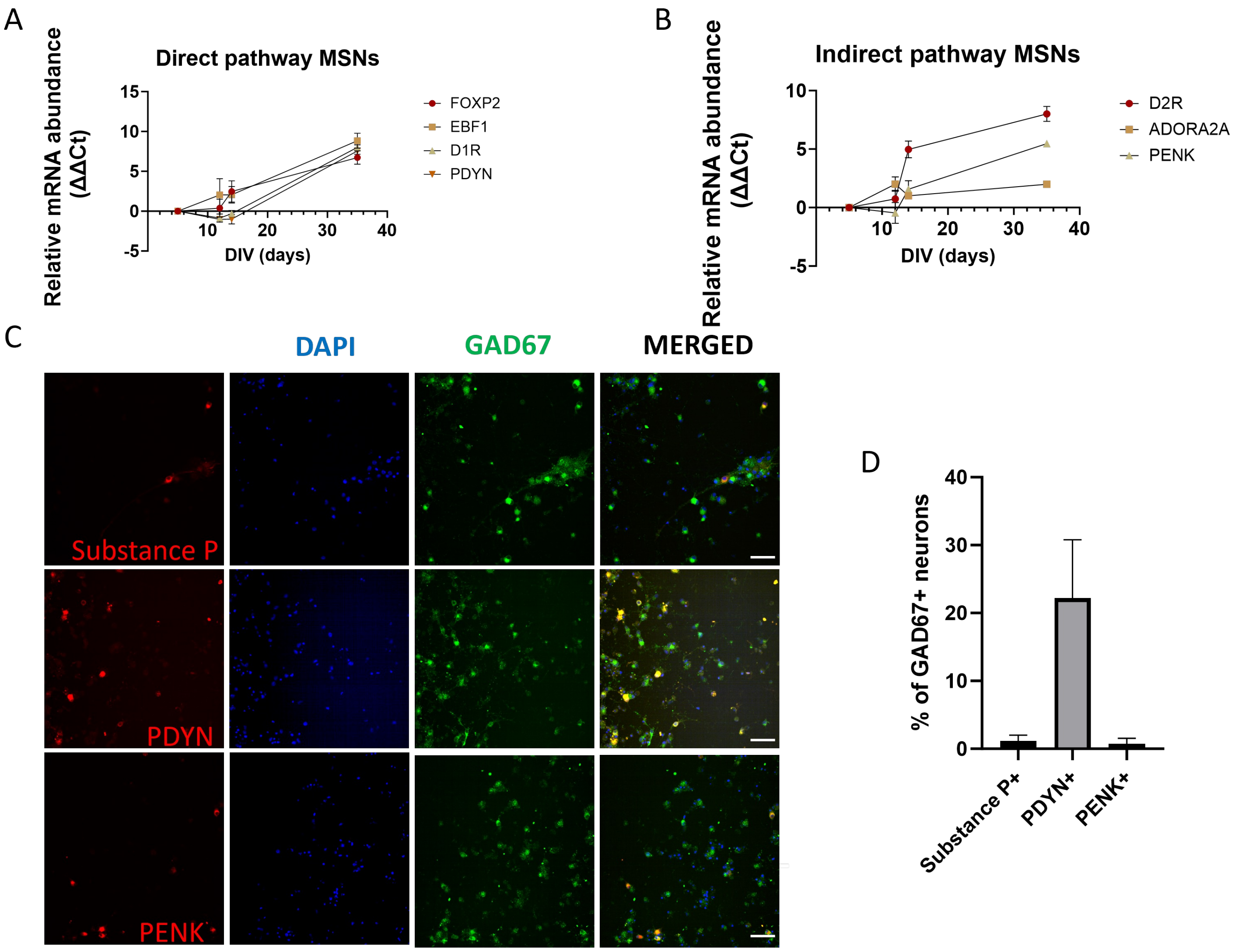

Supplementary Figure 3: iPSC-MSN cultures contained a mixture of both direct and indirect MSNs. (A-B) Transcript abundance of selected markers of (A) direct pathway MSNs and (B) indirect pathway MSNs over time. Expression was averaged from 2 differentiations of 3 independent iPSC lines. (C) Immunostaining for GAD67 (green), Substance P, or PENK or PDYN (red) at DIV 50. (D) Proportion of GAD67+ cells co-expressing different MSN subtype-specific neurotransmitters at DIV 50. All data are presented as mean ± sem, N = 2-3 differentiation experiments of 2-3 iPSC lines. Scale bar = 100 μm.

Sup. Fig. 4

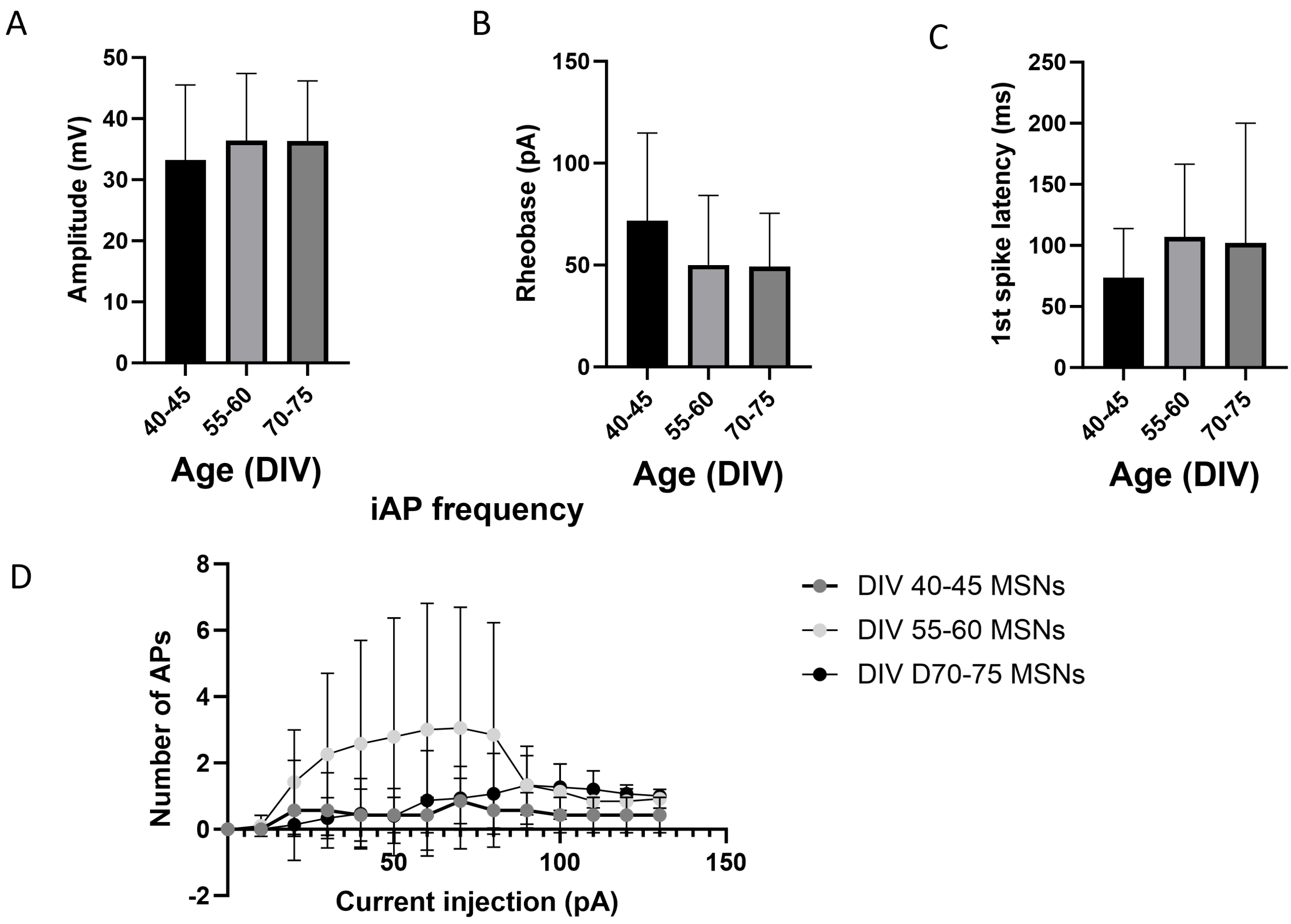

Supplementary Figure 4: Intrinsic electrophysiological properties of iPSC-MSNs in monocultures in terms of (A) rheobase, (B) evoked action potential amplitude, (C) time to first spike and (D) frequency of evoked action potentials. Data are presented as mean ± SD, from 2-3 differentiations of 3 different healthy control iPSC lines, one-way ANOVA test with Bonferroni correction test. ns p > 0.05.

Sup. Fig. 5

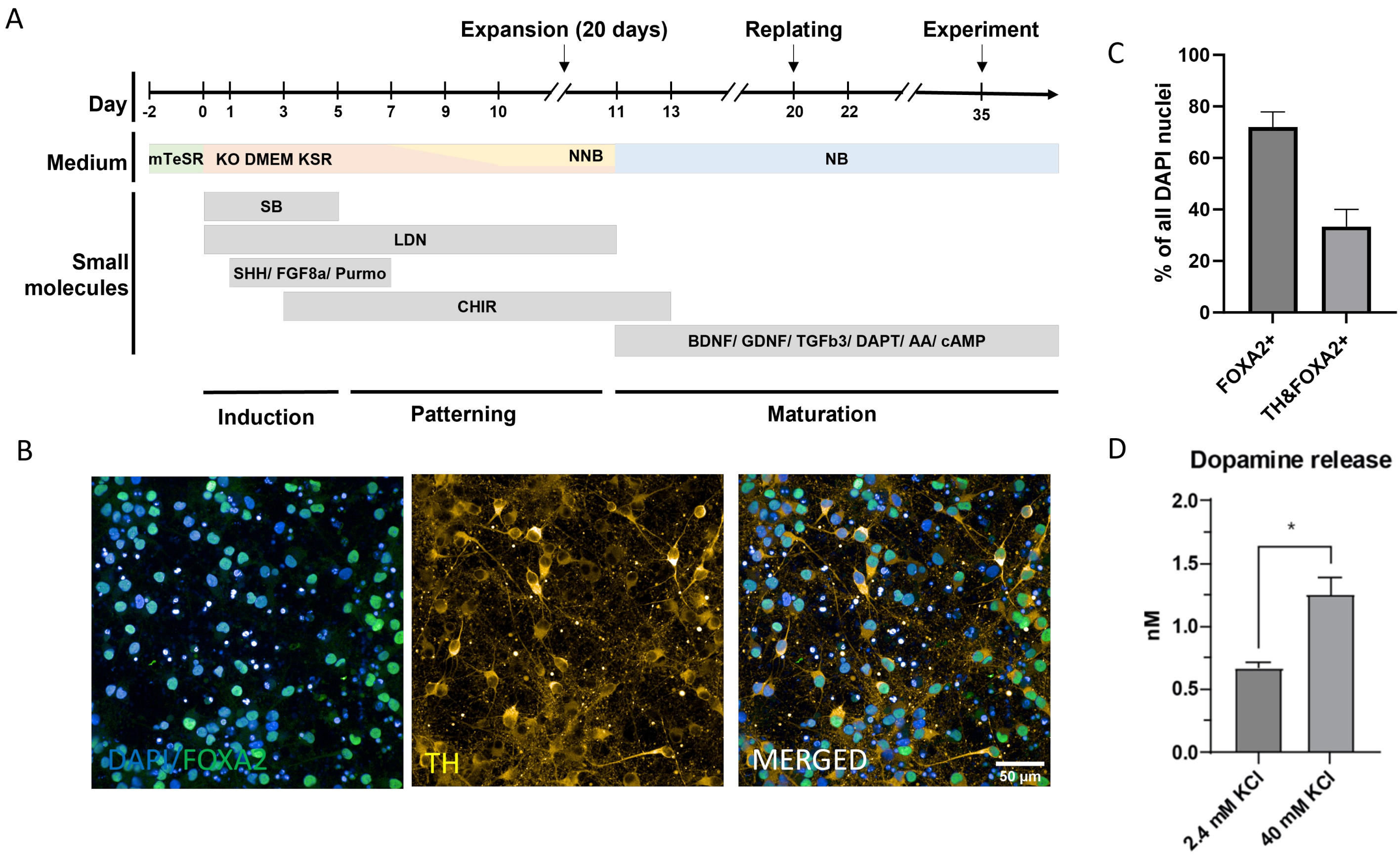

Supplementary Figure 5: Differentiation of DaNs from iPSCs. (A) Schematic of differentiation timeline of DaN from iPSCs. (B) Representative immunocytochemical images of iPSC-DaN cultures expressing FOXA2 (green) and TH (yellow). (C) Number of resulting neurons expressing canonical marker of midbrain origin, FOXA2, and of dopaminergic identity, TH. (D) Quantification of dopamine release by DIV 70 iPSC-DaN culture in tonic and stimulated (i.e. treatment with 40mM KCl for 5 minutes). Data are presented as mean ± sem, of 3 iPSC lines averaged from 2-3 differentiations. Student's t-test, * $p < 0.05$. Scale bar = 100 µm.

Sup. Fig. 6

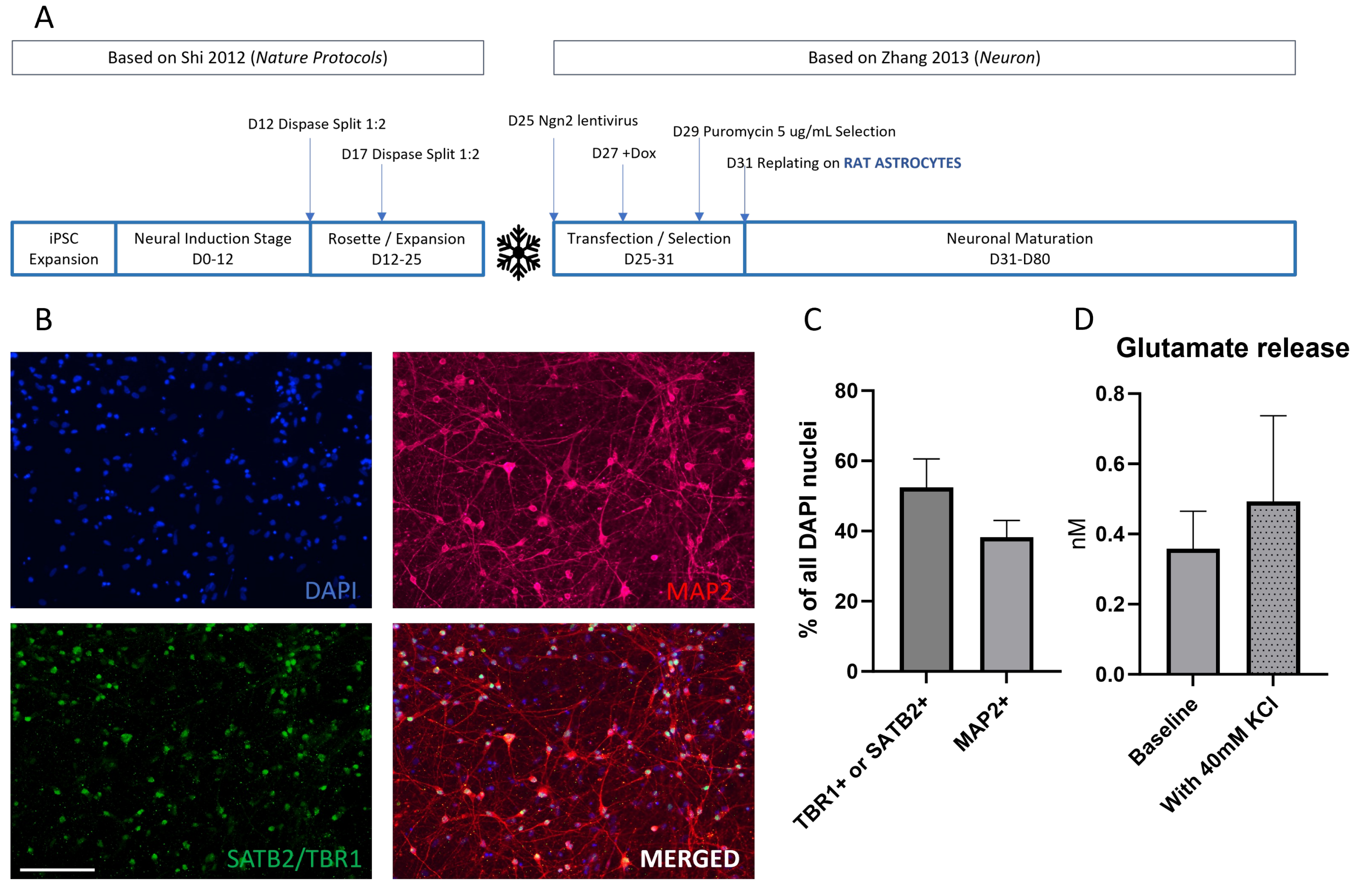

Supplementary Figure 6: Differentiation of CNs from iPSCs. (A) Schematic of differentiation timeline of CNs from iPSCs. (B) Representative fluorescent images of iPSC-CN cultures immune-stained exhibiting abundant expression of neuronal markers MAP2 (red) and layer-specific cortical markers SATB2 or TBR1 (green). (C) Percentage of neurons expressing either SATB2 or TBR1 in iPSC-CN cultures. (D) Quantification of glutamate concentration released by DIV 50 iPSC-CN cultures in basal and stimulated (i.e. treatment with 40mM KCl for 5 minutes) state. All data are presented as mean ± sem, of 3 healthy control iPSC lines. Student’s t test. Scale bar = 100 µm.

Sup. Fig. 7

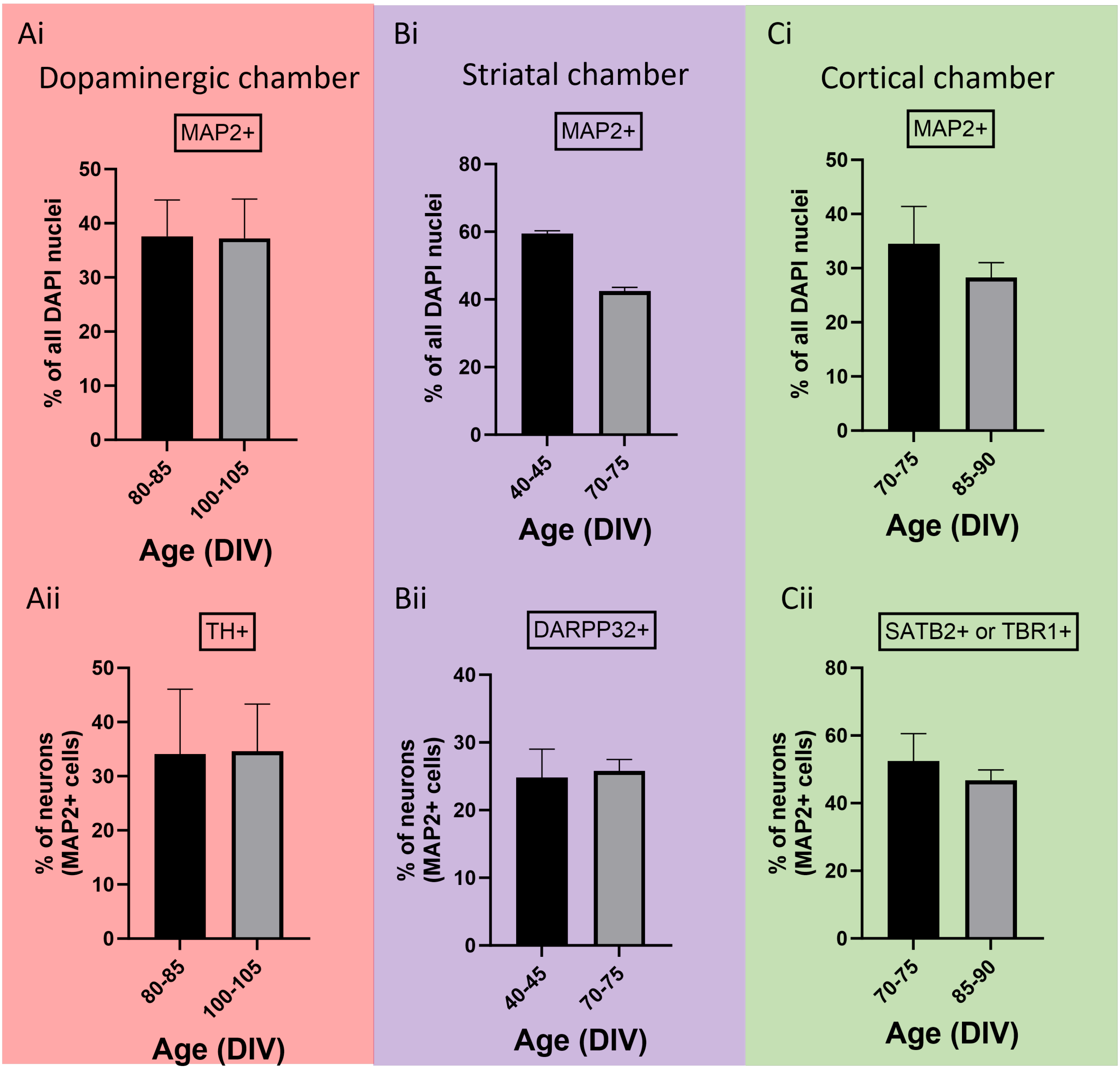

Supplementary Figure 7: Long-term viability of iPSC-neurons of cultured on three-chamber microfluidic device as characterised by (Ai, Bi, and Ci) the percentages of postmitotic neurons (i.e. MAP2+) within each chamber and (Aii, Bii, and CIii) the percentage of neurons expressing subtype-specific markers of the respective chamber over time. Data are presented as mean ± sem, of 3 healthy control iPSC lines. Student's t-test.

Sup. Fig. 8

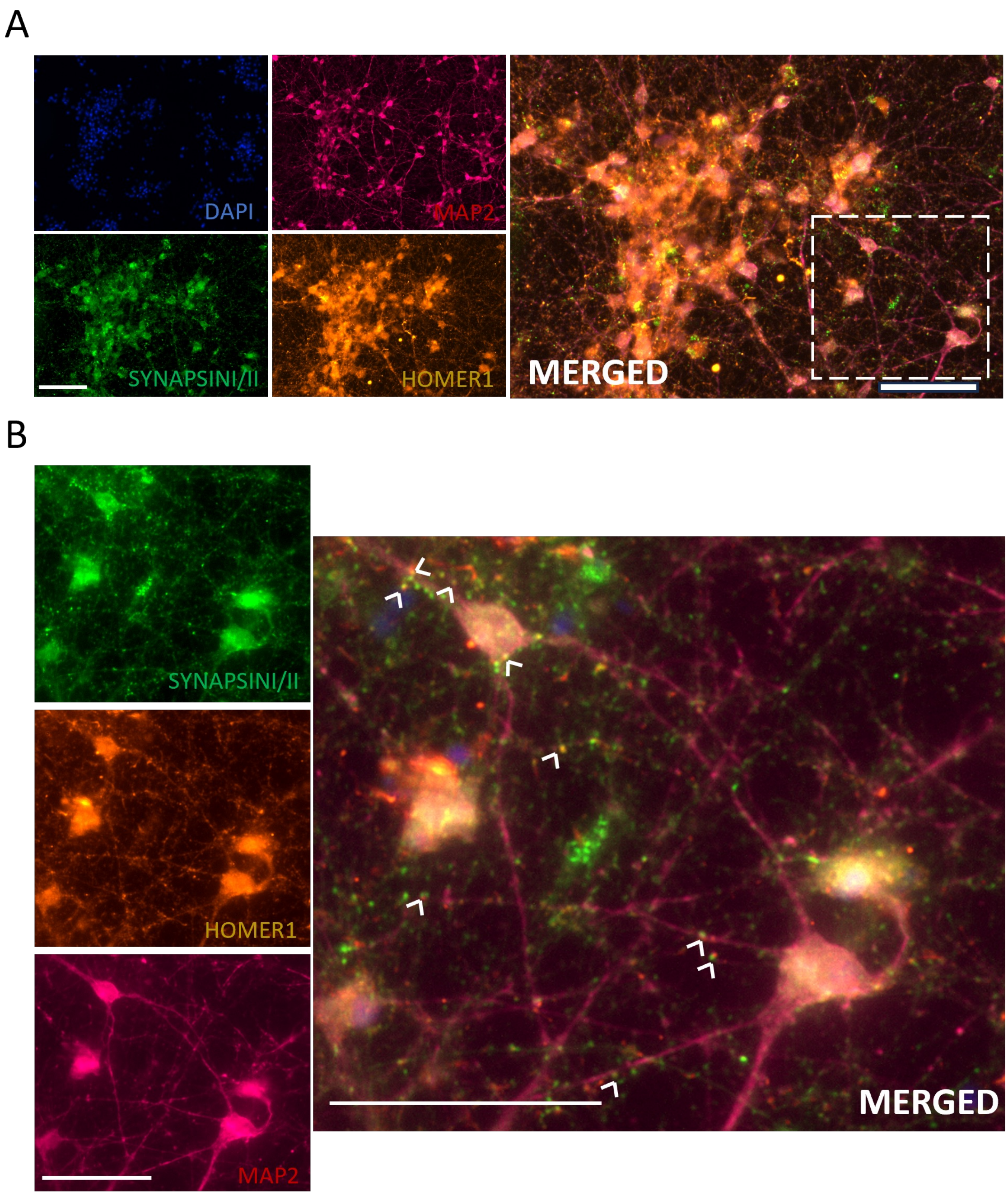

Supplementary Figure 8: Immunofluorescence images of DIV 40 iPSC-MSNs cultured on 3-chambered microfluidic devices with iPSC-CNs and iPSC-DaNs (A) and at higher magnification at 40x objective (B) showing presence of synapses labelled with presynaptic SynapsinI/II (green), postsynaptic Homer1 (yellow), and dendritic (MAP2) marker. Scale bar = 100 µm

Sup. Fig. 9

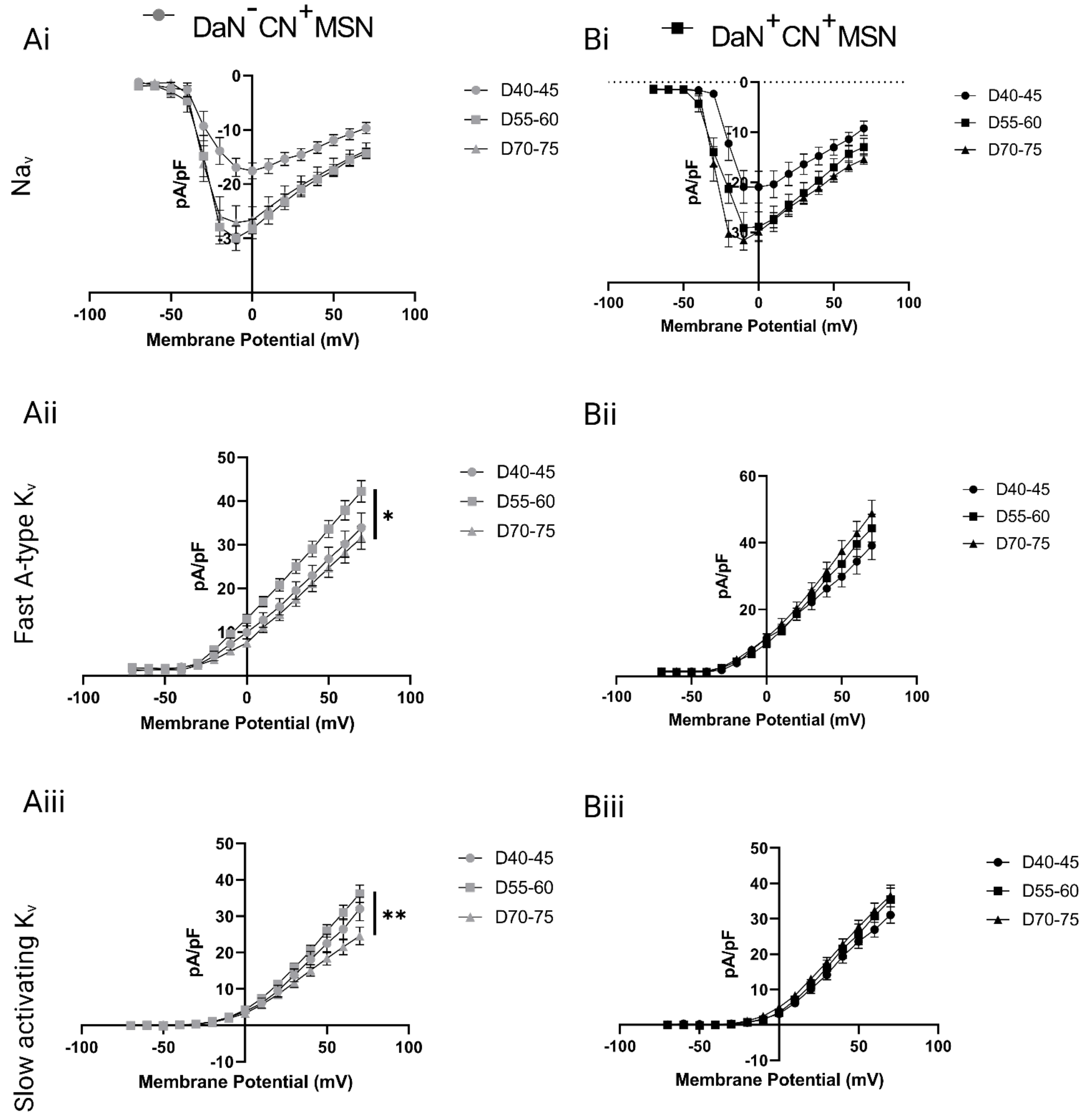

Supplementary Figure 9: Dopaminergic transmission mediated temporal changes of K_v current density. (A-B) Temporal profile of sodium and potassium ionic currents of cortico-striatal neurons in the (A) absence and (B) presence of dopaminergic input. Data are presented as mean ± SD, N=2 differentiation experiments of 3 iPSC lines, 2-way ANOVA with Bonferroni post-hoc test, *p<0.05, **p<0.001.

Sup. Fig. 10

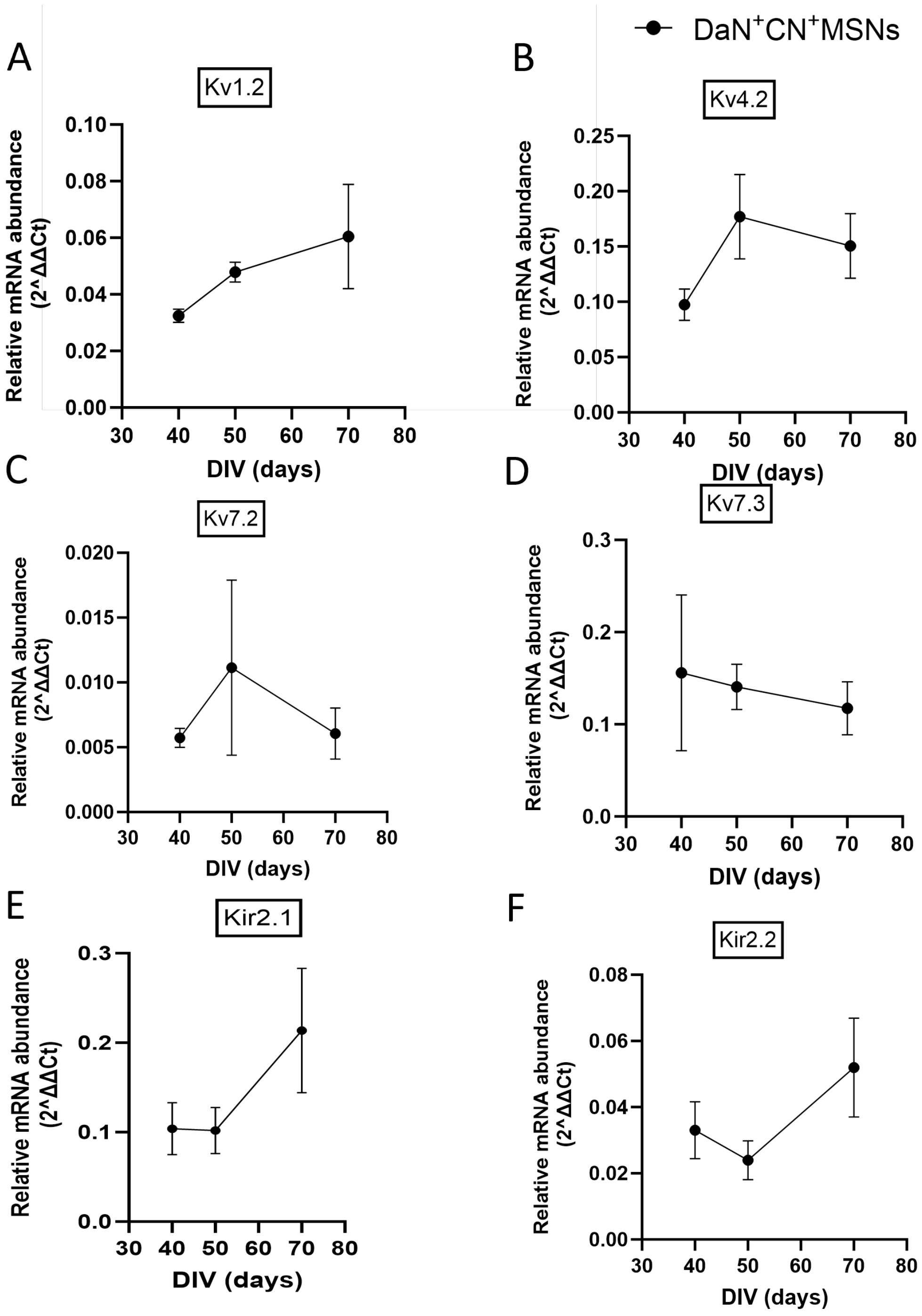

Supplementary Figure 10: Transcript expression of potassium channels in iPSC-MSNs in microcircuit with healthy control iPSC-DaNs (i.e.DaN$^+$CN$^+$ MSN) over time, including (A-D) voltage-gated potassium channels and (E, F) inwardly rectifying potassium channels. Relative expression of each channel transcript was normalised against expression level of housekeeping gene GAPDH, followed by relative expression of DARPP32 so as to account for potential variable expression of MSNs within different cultures. Data are presented as mean ± sem, of 3 healthy control iPSC lines, 1-way ANOVA with Bonferroni post-hoc test.

Sup. Fig. 11

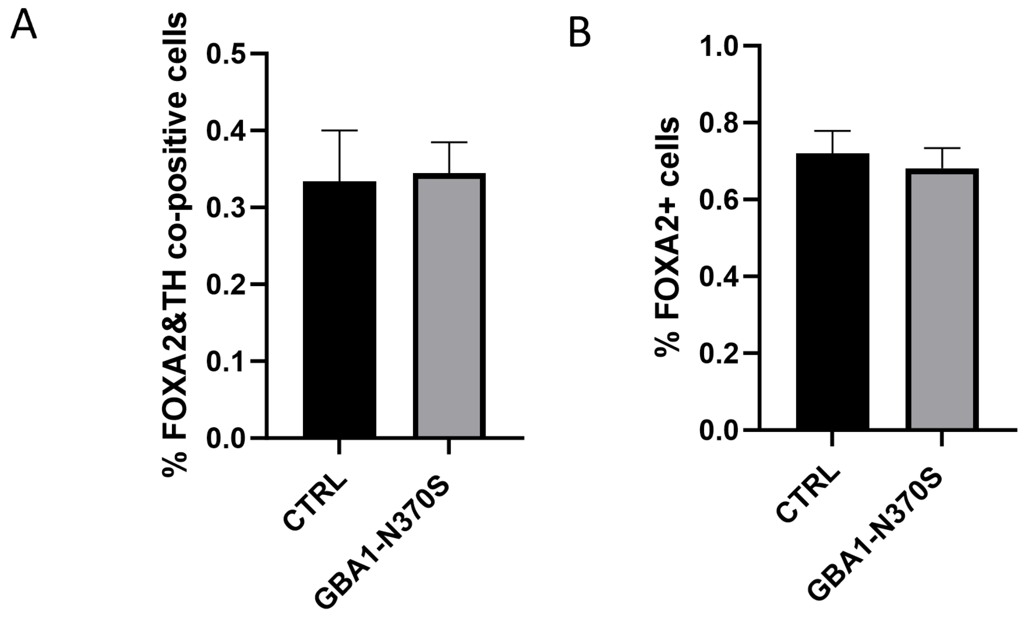

Supplementary Figure 11: Comparable levels of neurons expressing (A) TH and FOXA2 and (B) FOXA2 in healthy control-derived and *GBA-N370S* iPSC-DaN cultures at DIV 40. Percentage of positive cells were calculated against total DAPI nuclei count. Data are presented as mean ± sem, N=3 differentiation experiments of 3 iPSC lines, Student's t test, $p > 0.05$.

Sup. Fig. 12

A

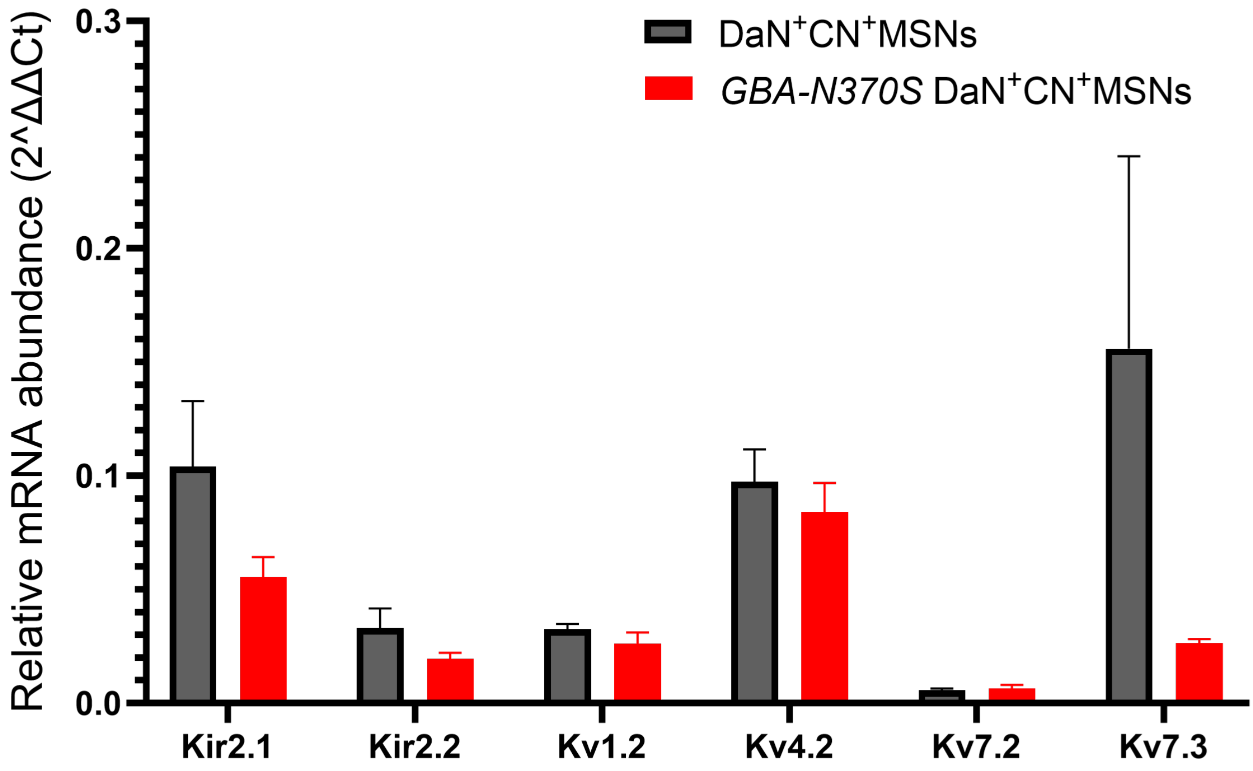

Supplementary Figure 12: Relative transcript abundance of potassium channels in iPSC-MSNs in microcircuit with either healthy control iPSC-DaNs (i.e.DaN$^+$CN$^+$ MSN) or *GBA-N370S* iPSC-DaNs (*GBA-N370S* DaN$^+$CN$^+$ MSN) at DIV 40-45. Data are presented as mean ± sem, of 3 healthy control iPSC lines, multiple Student's t-test.

Sup. Table 1

Supplementary Table 1: Table of evoked AP properties of iPSC-MSNs in monocultures over time.

	DIV 40-45	DIV 55-60	DIV 70-75
Rheobase (pA)	80.0±45.1	50.0±35.2	50.7±26.6
Amplitude (mV)	33.2±12.3	36.4±11.0	36.34±9.9
First spike latency (ms)	73.71±40.04	107.1±59.56	101.9±98.32

Supplementary Table 1: Table of evoked AP properties of iPSC-MSNs in monocultures over time.
