# Supplementary material for: Early deficits in an in vitro striatal microcircuit model carrying the Parkinson’s GBA-N370S mutation
Source: NPJ Parkinsons Dis. 2024 Apr 12;10:82. doi: 10.1038/s41531-024-00694-2 (PMC11014935; doi:10.1038/s41531-024-00694-2)
Supplement: Supplementary file 2 — Related Manuscript File [file 41531_2024_694_MOESM2_ESM.pdf]

nature portfolio

Corresponding author(s): Nora Bengoa-Vergniory

Last updated by author(s): Dec 29, 2023

Reporting Summary

Nature Portfolio wishes to improve the reproducibility of the work that we publish. This form provides structure for consistency and transparency in reporting. For further information on Nature Portfolio policies, see our Editorial Policies and the Editorial Policy Checklist.

Statistics

For all statistical analyses, confirm that the following items are present in the figure legend, table legend, main text, or Methods section.

n/a	Confirmed	
☐	☒	The exact sample size (*n*) for each experimental group/condition, given as a discrete number and unit of measurement
☐	☒	A statement on whether measurements were taken from distinct samples or whether the same sample was measured repeatedly
☐	☒	The statistical test(s) used AND whether they are one- or two-sided<br>*Only common tests should be described solely by name; describe more complex techniques in the Methods section.*
☐	☒	A description of all covariates tested
☐	☒	A description of any assumptions or corrections, such as tests of normality and adjustment for multiple comparisons
☐	☒	A full description of the statistical parameters including central tendency (e.g. means) or other basic estimates (e.g. regression coefficient) AND variation (e.g. standard deviation) or associated estimates of uncertainty (e.g. confidence intervals)
☐	☒	For null hypothesis testing, the test statistic (e.g. *F*, *t*, *r*) with confidence intervals, effect sizes, degrees of freedom and *P* value noted<br>*Give P values as exact values whenever suitable.*
☒	☐	For Bayesian analysis, information on the choice of priors and Markov chain Monte Carlo settings
☒	☐	For hierarchical and complex designs, identification of the appropriate level for tests and full reporting of outcomes
☒	☐	Estimates of effect sizes (e.g. Cohen's *d*, Pearson's *r*), indicating how they were calculated

Our web collection on statistics for biologists contains articles on many of the points above.

Software and code

Policy information about availability of computer code

Data collection	Commercial imaging software associated with Olympus FluoView FV1000, the Opera Phenix High-content Screening system (PerkinElmer) or the Invitrogen EVOS ™ FL Auto (ThermoFisher) were used<br>Electrophysiological data were acquired with MultiClamp 700B amplifier (Molecular Devices) and Digidata 1550B digitiser (Molecular Devices)<br>Colourimetric readouts were acquired using the PHERAstar system<br>Exact usage of data collection could be found in the Method section
Data analysis	Images were processed using Harmony (Perkin Elmer; RRID:SCR_018809), CellProfiler (RRID:SCR_007358) , Imaris 9.6.0 (Bitplane, South Windsor, CT, USA, RRID:SCR_007370), or the FIJI distribution of the open source image processing application ImageJ (RRID:SCR_003070)<br>All statistical analyses were performed on GraphPad Prism 10 (GraphPad Software, RRID:SCR_002798).<br>Electrophysiological analysis were performed on Clampfit 10.7 (RRID:SCR_011323).<br>Details of exact usage as well as data availability is provided in the Method section

For manuscripts utilizing custom algorithms or software that are central to the research but not yet described in published literature, software must be made available to editors and reviewers. We strongly encourage code deposition in a community repository (e.g. GitHub). See the Nature Portfolio guidelines for submitting code & software for further information.

Data

Policy information about availability of data

All manuscripts must include a data availability statement. This statement should provide the following information, where applicable:

- Accession codes, unique identifiers, or web links for publicly available datasets
- A description of any restrictions on data availability
- For clinical datasets or third party data, please ensure that the statement adheres to our policy

The data that support the findings of this study are deposited on Zenodo (DOI 10.5281/zenodo.7661355). All details of the primers, antibodies, cell lines, and software used in this work are available on Zenodo (DOI 10.5281/zenodo.7661355). Protocols associated with this work can be found on protocols.io (DOI 10.17504/protocols.io.x54v9dw61g3e/v1).

Research involving human participants, their data, or biological material

Policy information about studies with human participants or human data. See also policy information about sex, gender (identity/presentation), and sexual orientation and race, ethnicity and racism.

Reporting on sex and gender	Findings in this study do not apply to a specific sex or gender. The cell lines used in this study is relative gender balanced, including 3 healthy controls and 3 GBA N370S PD lines.
Reporting on race, ethnicity, or other socially relevant groupings	All lines used in this study is ethnically matched to be of Caucasian, non-Hispanic origin.
Population characteristics	The lines used in this study are relatively poorly age-matched: controls were 72, 75, and 78 and GBAs were 51, 46 and 81. We acknowledge the disparity in the age and have included this information in Table 1 of the paper, we believe this is a common limitation in stem cell research of which there are plenty of published examples
Recruitment	Information not available
Ethics oversight	Ethics committee: National Health Service, Health Research Authority, NRES Committee South Central, Berkshire, UK, REC 10/H0505/71

Note that full information on the approval of the study protocol must also be provided in the manuscript.

Field-specific reporting

Please select the one below that is the best fit for your research. If you are not sure, read the appropriate sections before making your selection.

☒ Life sciences ☐ Behavioural & social sciences ☐ Ecological, evolutionary & environmental sciences

For a reference copy of the document with all sections, see nature.com/documents/nr-reporting-summary-flat.pdf

Life sciences study design

All studies must disclose on these points even when the disclosure is negative.

Sample size	No predetermined sample size calculation was performed and sample size were determined by common practice in the field and based on previous studies in the field.
Data exclusions	No data which met the quality criteria of the designed experiments (e.g. access resistance) was excluded in this study.
Replication	All key experiments were replicated at least twice and number of replicates were mentioned in figure legends.
Randomization	Cell cultures were randomly selected from culturing batches for all experiments
Blinding	This study involved unbiased analysis and quantification for immunostaining, transcript expression, electrophysiological properties. Data collection and analysis were performed using unbiased and common pipelines.

Reporting for specific materials, systems and methods

We require information from authors about some types of materials, experimental systems and methods used in many studies. Here, indicate whether each material, system or method listed is relevant to your study. If you are not sure if a list item applies to your research, read the appropriate section before selecting a response.

Materials & experimental systems

n/a	Involved in the study
☐	☒ Antibodies
☐	☒ Eukaryotic cell lines
☒	☐ Palaeontology and archaeology
☒	☐ Animals and other organisms
☒	☐ Clinical data
☒	☐ Dual use research of concern
☒	☐ Plants

Methods

n/a	Involved in the study
☒	☐ ChIP-seq
☒	☐ Flow cytometry
☒	☐ MRI-based neuroimaging

Antibodies

Antibodies used

All details of the antibodies used in this work are available on Zenodo (DOI 10.5281/zenodo.7661355).

Validation

All antibodies have been validated by the supplying company. Mentioning of these anntibodies in open-access scientific literature was detailed using RRID search.

Eukaryotic cell lines

Policy information about cell lines and Sex and Gender in Research

Cell line source(s)

The iPS cell lines have been previously characterized: 3 control iPSC lines SFC067-03-01 (RRID:CVCL_RD75), SFC156-03-01 (EBiSC Cat# STBCi101-A, RRID:CVCL_RD71) and SFC856-03-04 (RRID:CVCL_RC81), 3 GBA-N370S PD lines MK082-26 (RRID:CVCL_IJ04), MK088-01 (EBiSC Cat# UOXFi003-A), and MK071-03 (EBiSC Cat# UOXFi001-B)

Authentication

The OPDC Discovery cohort was supported by the National Institute for Health Research (NIHR) Oxford Biomedical Research Centre based at Oxford University Hospitals NHS Trust and University of Oxford and the Dementia and Neurodegenerative Diseases Research Network (DeNDRoN)

Mycoplasma contamination

All cell lines were routinely tested negative for mycoplasma contamination

Commonly misidentified lines
(See ICLAC register)

No commonly misidentified lines were used in this study.

Plants

Seed stocks

n/a

Novel plant genotypes

n/a

Authentication

n/a
